# Supplementary material for: Electrocatalytic CO2 reduction to ethylene in an acid-fed membrane electrode assembly at 10 A
Source: Nat Commun. 2025 Nov 28;16:10783. doi: 10.1038/s41467-025-65831-8 (PMC12663450; doi:10.1038/s41467-025-65831-8)
Supplement: Supplementary file 1 — Supplementary Information [file 41467_2025_65831_MOESM1_ESM.pdf]

Supplementary Information

Electrocatalytic CO_2 Reduction to Ethylene in An Acid-Fed Membrane Electrode Assembly at 10 A

Derong Chen[1,2,3 #], Jia Liu[2, #], Yijia Yuan[2, #], Xiaocang Han[2], Kun Zhang[2], Qikun Hu[2], Shuhe Han[4], Shibo Xi[5], Quan-Hong Yang[1,3*], and Kian Ping Loh[2,6*]

[#]These authors contributed equally to this work. Correspondence and requests for materials should be addressed Q.-H. Yang (qhyangcn@tju.edu.cn) and K. P. Loh (chmlohkp@nus.edu.sg)

[1] Joint School of the National University of Singapore and Tianjin University, International Campus of Tianjin University, Binhai New City, Fuzhou 350207, China

[2] Department of Chemistry, National University of Singapore, 3 Science Drive 3, Singapore 117543, Singapore

[3] Nanoyang Group, Tianjin Key Laboratory of Advanced Carbon and Electrochemical Energy Storage, School of Chemical Engineering and Technology, and Collaborative Innovation Centre of Chemical Science and Engineering (Tianjin), Tianjin University, Tianjin 300072, China

[4] Department of Applied Physics, The Hong Kong Polytechnic University, Hung Hom, Kowloon, Hong Kong, China

[5] Institute of Sustainability for Chemicals, Energy and Environment, A*STAR, Singapore

[6] Centre for Hydrogen Innovations, National University of Singapore, E8, 1 Engineering Drive 3, 117580 Singapore

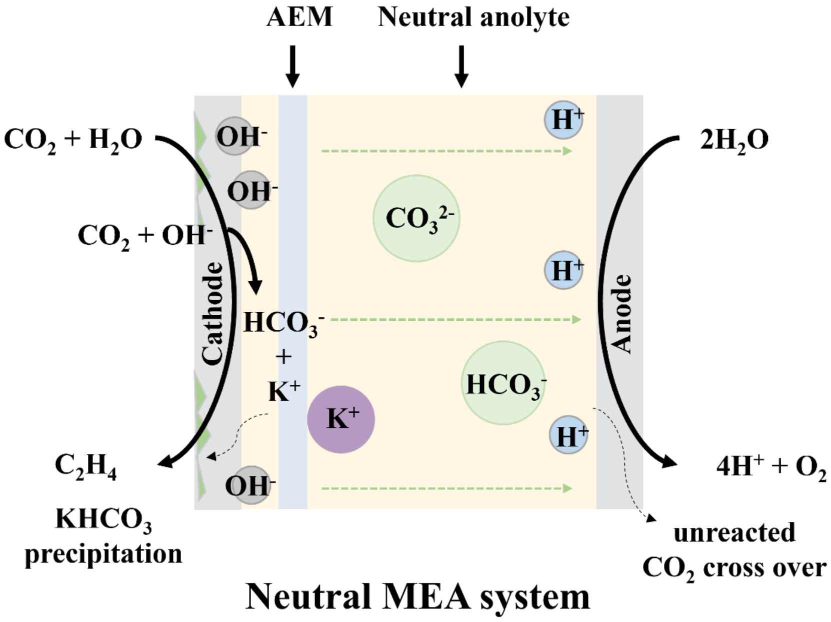

Supplementary Fig. 1 | Schematic of the neutral MEA system.

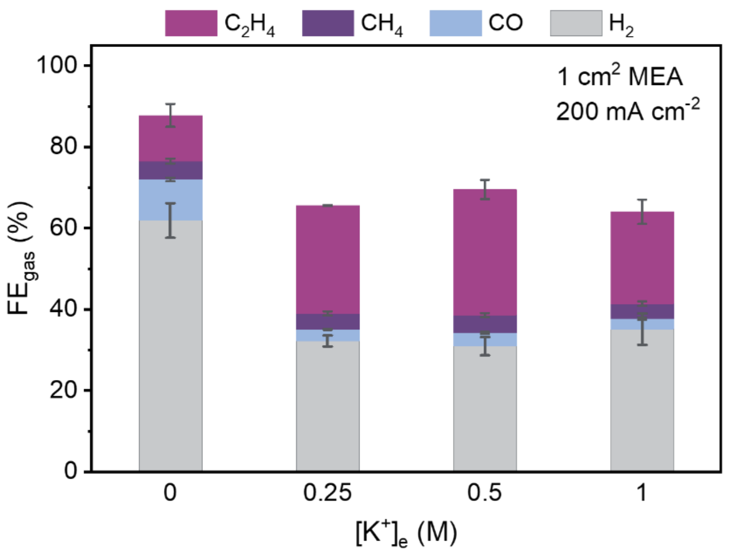

Supplementary Fig. 2 | The CO_2R gas product distribution in AEM-based MEA system in H_2SO_4 with different K_2SO_4 concentrations at 200 mA cm^{-2} (n = 3 replicates). $[K^+]_e$ was denoted as different K^+ concentration (from 0 M to 1 M) in bulk anolyte (H_2SO_4/K_2SO_4; pH~2). No iR correction was applied. Source data for Supplementary Fig. 2 are provided as a Source Data file.

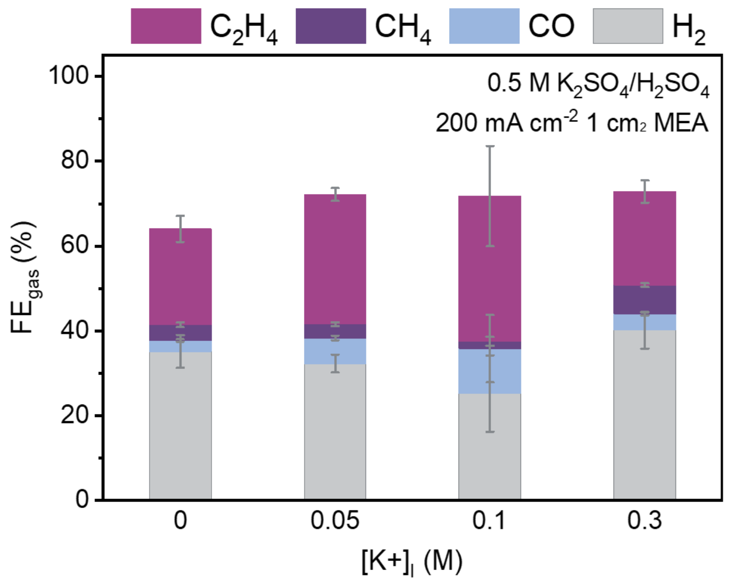

Supplementary Fig. 3 | The CO_2R gas product distribution in AEM-based MEA system in H_2SO_4 with different local concentrations of K^+ at 200 mA cm^{-2} (n = 3 replicates). $[K^+]_I$ was denoted as different interfacial K^+ concentration (from 0 M to 0.3 M) in bulk anolyte (H_2SO_4 with 0.5 M K_2SO_4; pH~2). No iR correction was applied. Source data for Supplementary Fig. 3 are provided as a Source Data file.

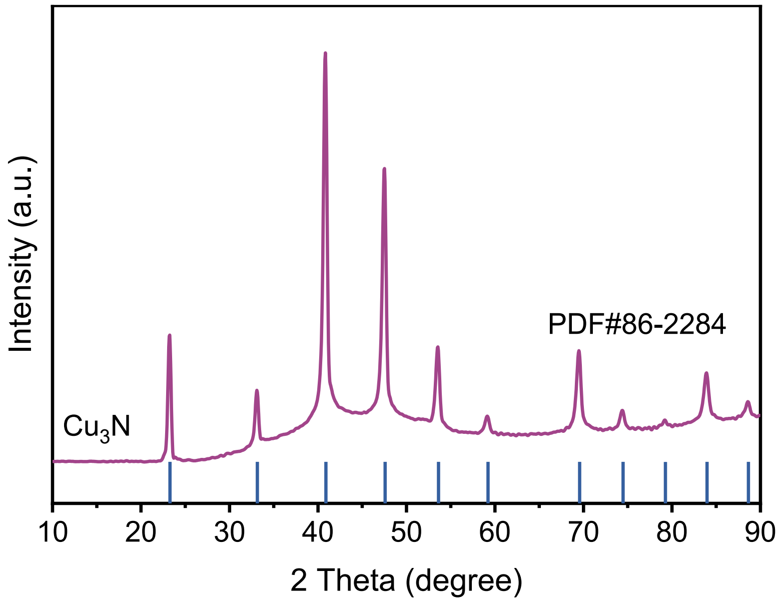

Supplementary Fig. 4 | XRD pattern of synthesized Cu_3N. The PXRD pattern of Cu_3N exhibits intense peaks at 23.27, 40.89, and 47.57°, which correspond to the (100), (111), and (200) facets, respectively. Powder X-ray diffraction (PXRD) was used to prove the pure phase of Cu_3N. Source data for Supplementary Fig. 4 are provided as a Source Data file.

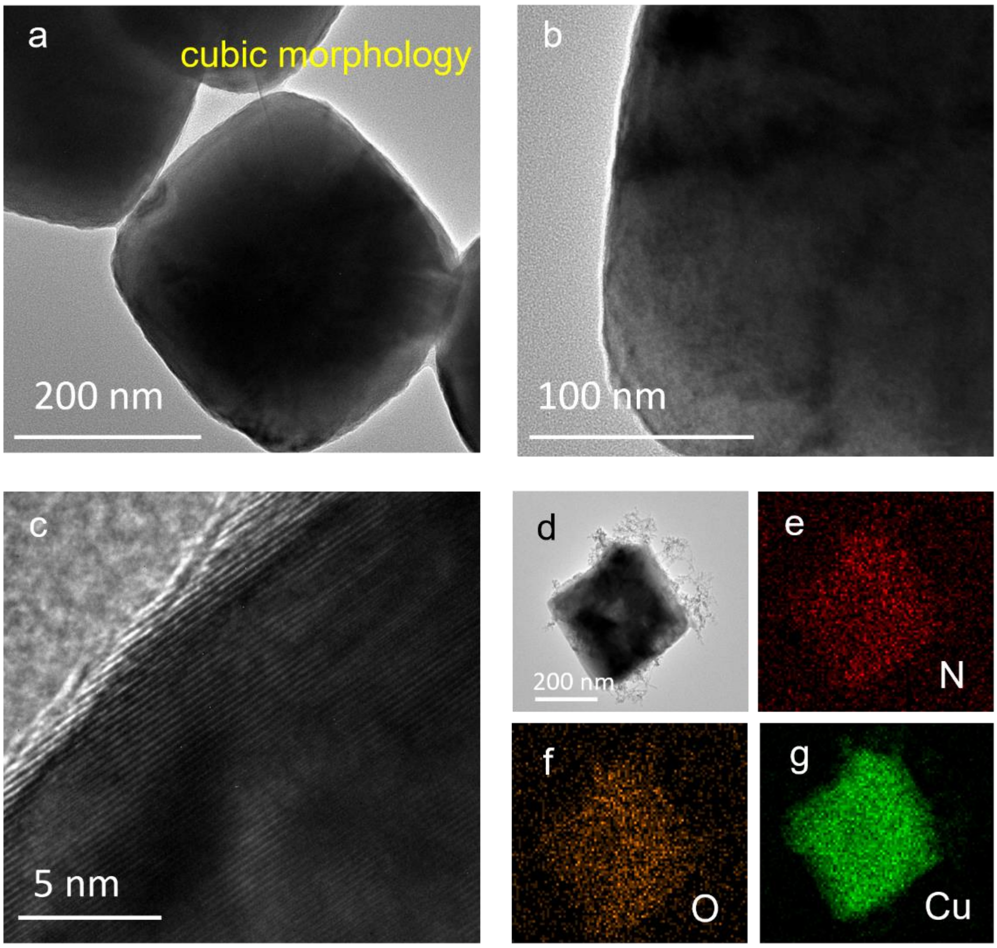

Supplementary Fig. 5 | TEM images (a-c) and corresponding EDS element mapping (d-g) of Cu_3N catalyst.

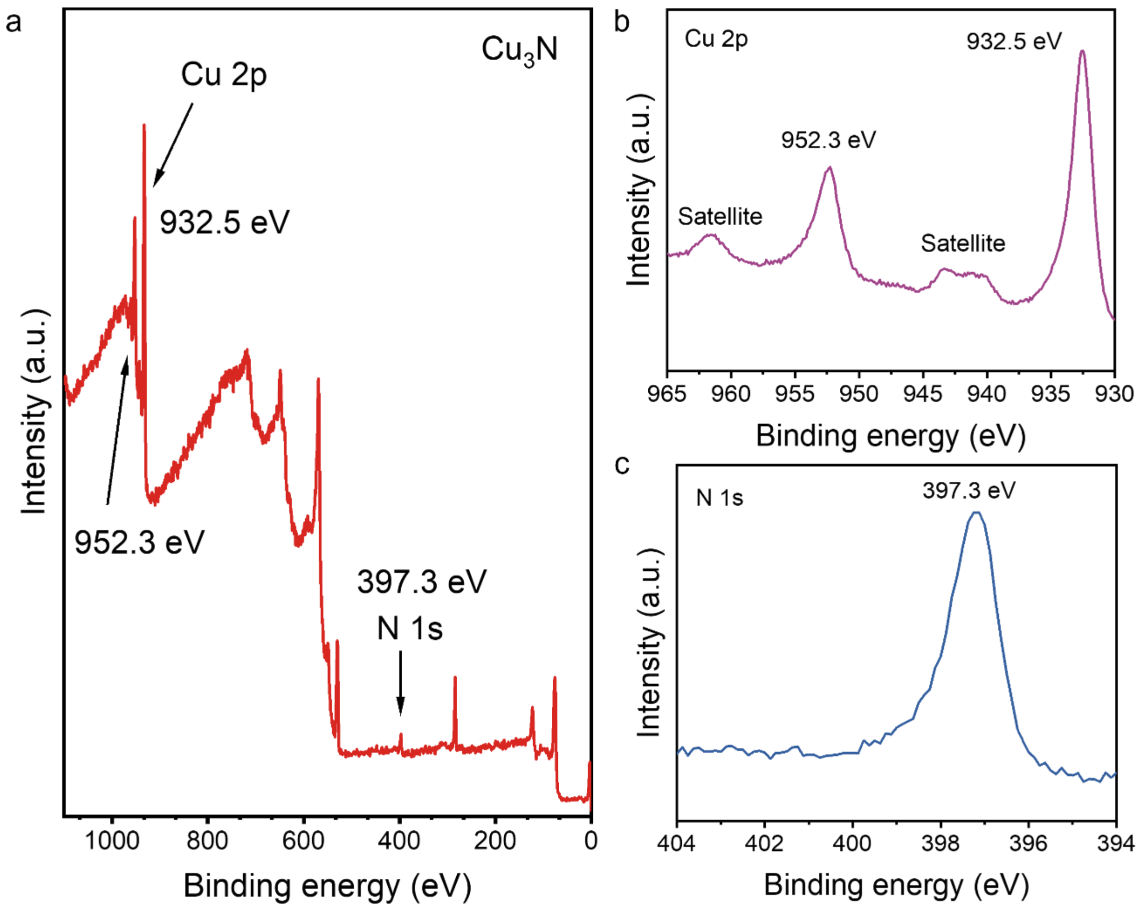

Supplementary Fig. 6 | XPS patterns of Cu 2p (b) and N 1s (c) spectra of Cu_3N catalyst. X-ray photoelectron spectroscopy of Cu atom in Cu_3N catalyst showed an evident peak at 952.3 eV and 932.5 eV split spin-orbit components in the Cu_{2p} spectrum. the peak of covalent N was detected at a binding energy of 397.3 eV. Source data for Supplementary Fig. 6 are provided as a Source Data file.

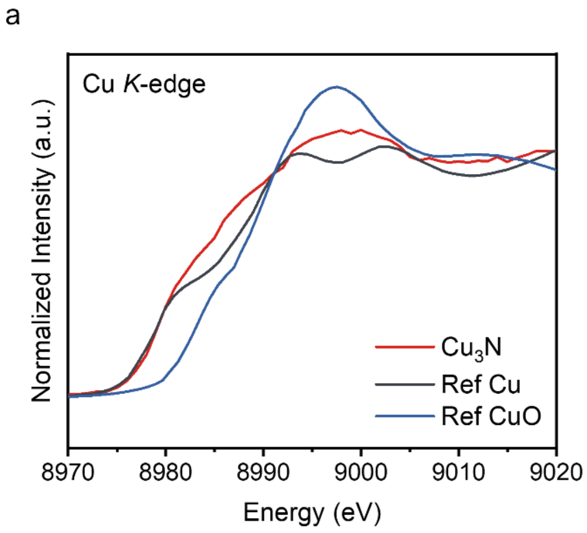

Supplementary Fig. 7 | Cu Kedge XANES spectra (a) and FT-EXAFS spectra (b) of Cu_3N, reference Cu, and reference CuO. Extended X-ray absorption fine structure (EXAFS) spectroscopy confirmed the existence of the Cu-N bonding located at 1.34 Å in Cu_3N catalyst and the Cu-Cu coordination at 2.33 Å was observed in the FT-EXAFS spectra. Source data for Supplementary Fig. 7 are provided as a Source Data file.

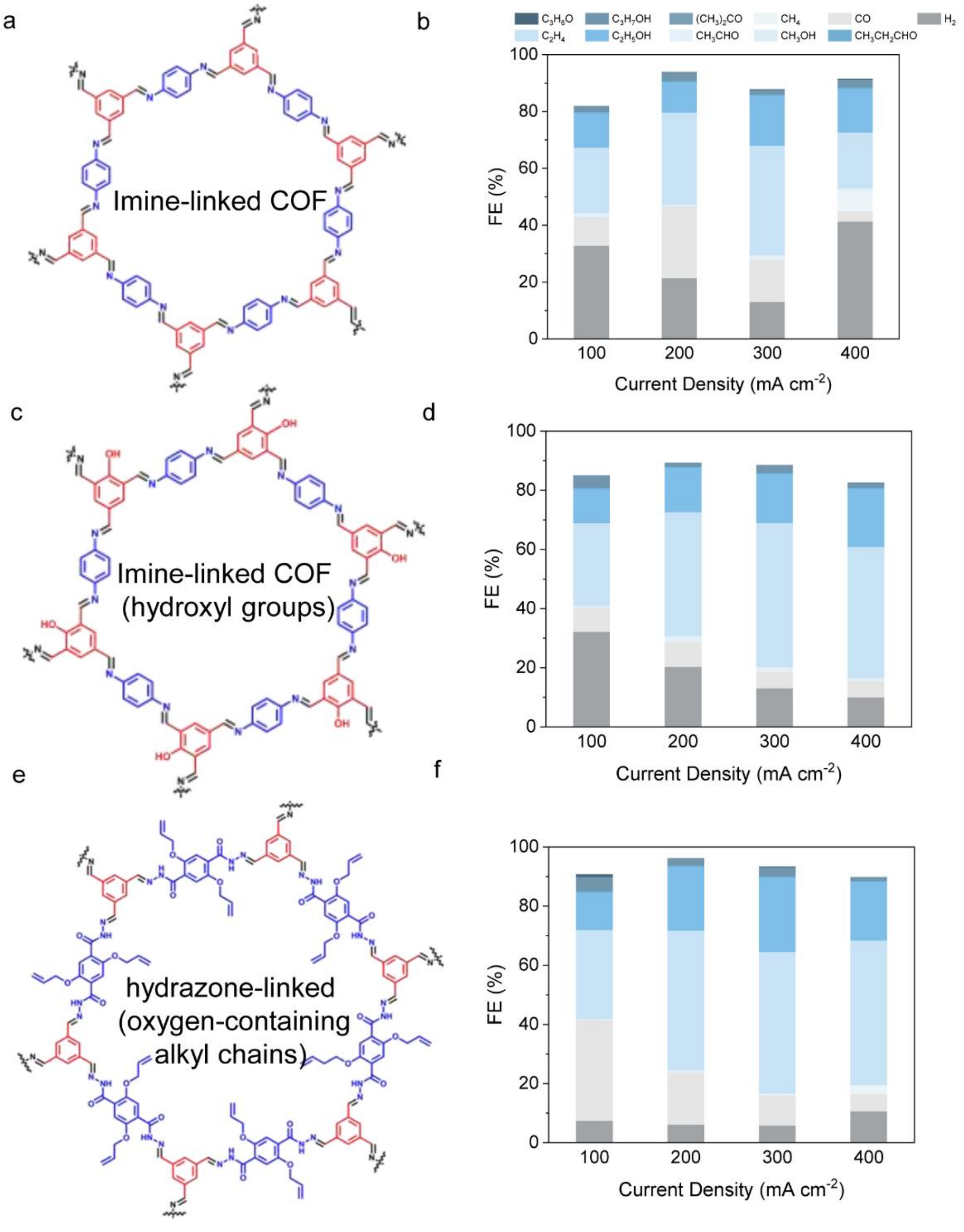

Supplementary Fig. 8 | Molecular structures of COF group and CO_2R product distribution in the acid-fed COF-modified AEM MEA system at different current density in 0.5 M K_2SO_4/H_2SO_4 (pH~2) condition. We first implemented an imined-linked COF (Fig. a) in an acidic MEA system, achieving a FE of 38.6 % for C_2H_4 at 300 mA cm^{-2}. However, HER dominated at higher current densities, with FE_{H_2} reaching approximately 40%. To achieve better performance for the acid-fed MEA system, we synthesized and screened acid-resistant COFs with tailored reticular backbones and functional groups. As shown in Figs. c and e, we introduced hydroxyl groups (Fig. c) or oxygen-containing alkyl chains (Fig. e) on COF backbones at the molecular level. This approach systematically optimized the hydrophilic-hydrophobic balance of the materials.

Unlike hydroxyl groups, the three-dimensional structure of oxygen-containing alkyl chains introduces steric hindrance that impedes water molecules from approaching the oxygen atoms. This kinetic barrier markedly reduces the efficiency of hydrogen bond formation with water molecules. Consequently, the COF material in Fig. e exhibits strong hydrophobicity. The formation of a dynamic hydrophobic layer on the surface facilitates rapid diffusion of non-polar CO_2 molecules while also impeding proton migration via steric hindrance. Moreover, the incorporation of oxygen-containing alkyl chains establishes an in-plane hydrogen bonded network within the 2D COF framework. This supramolecular interaction enhances interlayer π-π stacking regularity and boosts crystallinity through non-covalent bond constraints. It also creates directional nanochannels that facilitate substance transport, resulting in increased C_2H_4 selectivity ($FE_{C_2H_4}$: 48.9%) and suppressed HER (FE_{H_2}: 10.5%) at 400 mA cm^{-2}. No iR correction was applied. Source data for Supplementary Figs. 8b, d, and f are provided as a Source Data file.

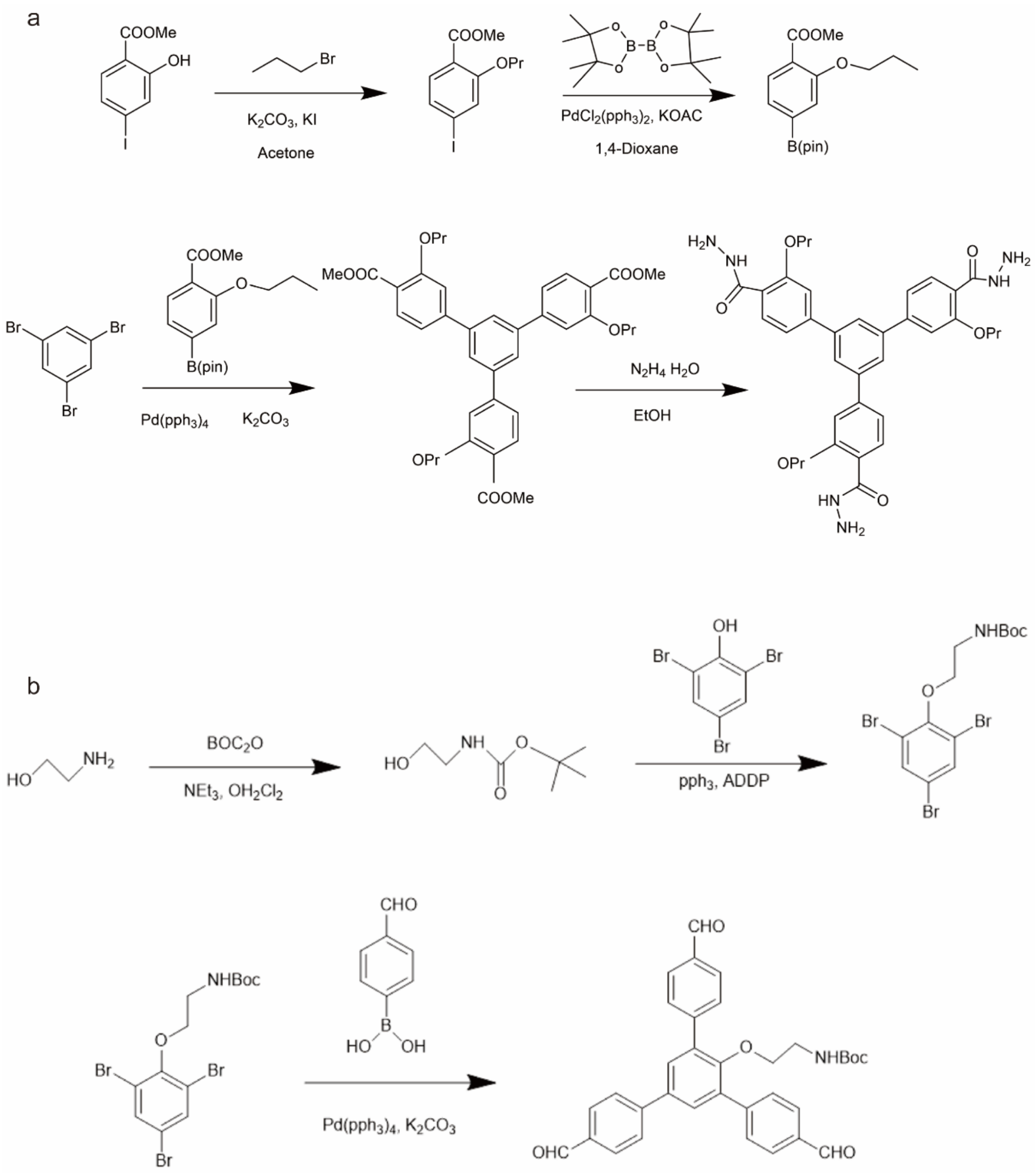

Supplementary Fig. 9 | Synthesis of ThzOPr and TFPBr. (a) ThzOPr was prepared following a reported method: methyl 2-hydroxy-4-iodobenzoate was alkylated with 1-propylbromide in acetone under reflux, borylated with bis(pinacolato)diboron, and coupled with 1,3,5-tribromobenzene, followed by hydrazinolysis to afford ThzOPr (68%). (b) TFPBr was synthesized via a three-step sequence: ethanolamine was Boc-protected, the intermediate was coupled with 2,4,6-tribromophenol using triphenylphosphine/azodicarbonyl dipiperidine, and finally reacted with 4-formylphenylboronic acid under Pd-catalyzed conditions to give TFPBr. Th-TF COF was obtained by solvothermal condensation of 1,3,5-triformylbenzene with the amine precursor in 1,4-dioxane and acetic acid at 120 °C for 3 days, yielding the hydrazone-linked framework after solvent exchange and vacuum drying.

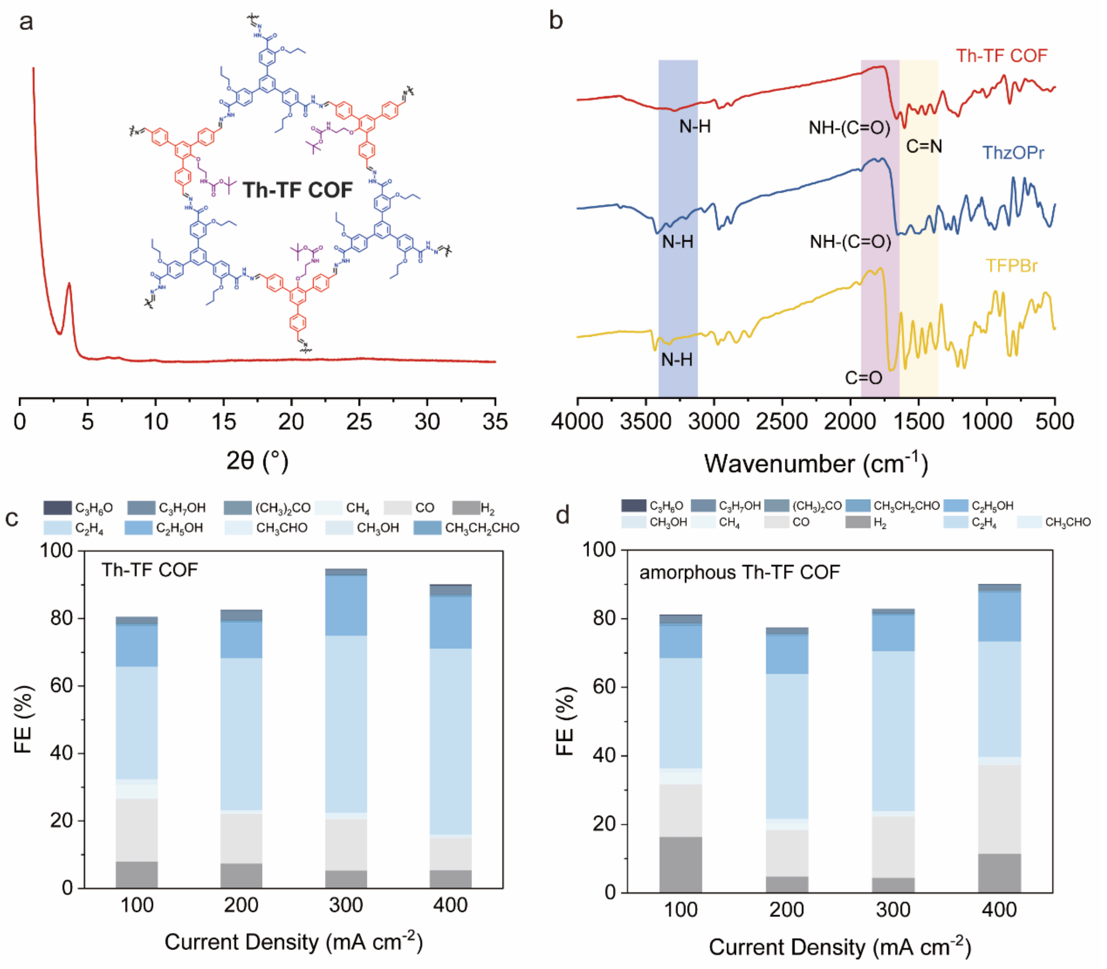

Supplementary Fig. 10 | Based on the screening results in Supplementary Fig. 8, oxygen-containing alkyl chains were incorporated into the hydrazone-linked backbone to yield Th-TF COF. This molecular engineering strategy enhances charge polarization within the framework, creating local protonation channels. Structural characterization further confirmed the framework formation: (a) PXRD pattern showing a low-angle (100) reflection at 2θ ≈ 3.67°; a weak hump near 25° suggests π–π stacking but remains unresolved due to limited crystallinity. (b) FTIR spectra of Th-TF COF and its precursors, highlighting bands at 1598 cm^{-1} (C=N), 1667 cm^{-1} (C=O), and 3285 cm^{-1} (N-H). Red-shift and attenuation relative to the precursors confirm conjugation and near-complete linkage formation. When implemented in an acid-fed MEA, Th-TF COF exhibits dual regulatory functions: facilitating K^+ transport through potential drops at the catalyst–COF interface, and promoting OH^- migration through H-bond interactions in the COF channels. In situ measurements and MD simulations (Figs. 4 and 5) corroborate these effects, which collectively enable excellent catalytic performance, achieving a FE of 55% for

C_2H_4 and 75% for C_{2+} products at 400 mA cm^{-2} (pH ~2, 1 M K^+). No iR correction was applied. Source data for Supplementary Fig. 10 are provided as a Source Data file.

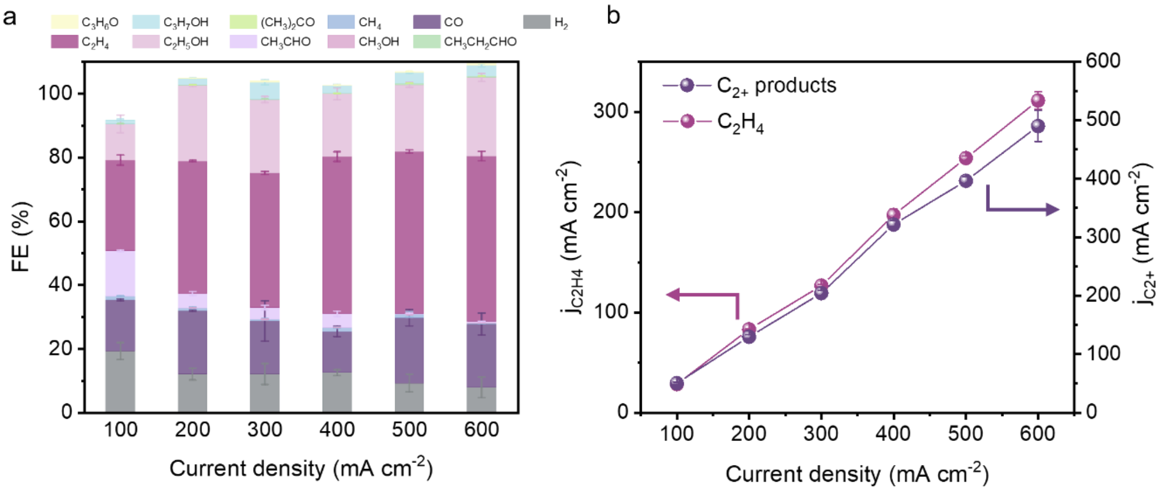

Supplementary Fig. 11 | CO_2R product distribution (a) and C_2H_4, C_{2+} products partial current of CNCP electrodes at different current densities in acid-fed MEA system in 0.5 M K_2SO_4/H_2SO_4 (ph~1) condition. No iR correction was applied. Source data for Supplementary Fig. 11 are provided as a Source Data file.

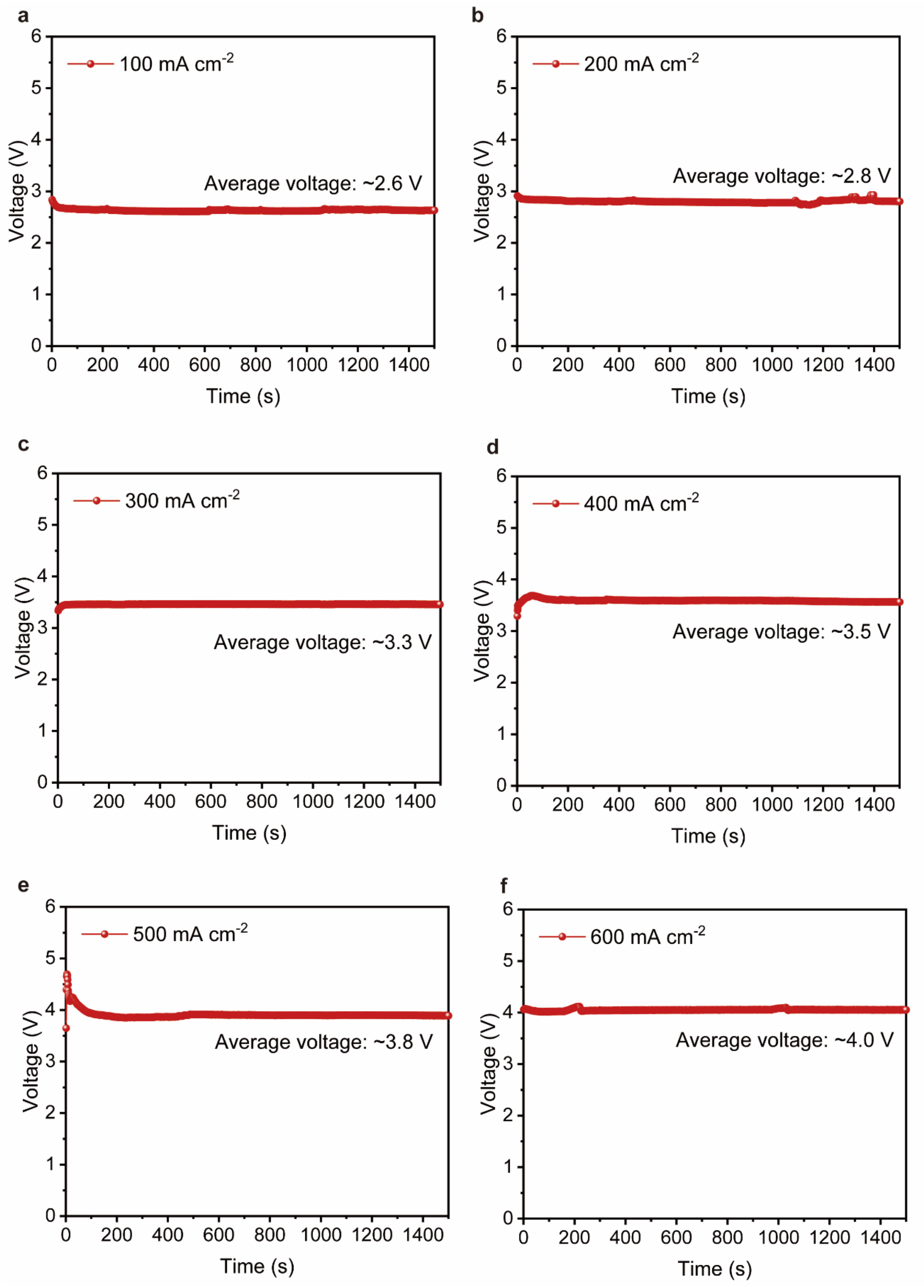

Supplementary Fig. 12 | The V-t curves of CO_2R using CNCP catalyst in 0.5 M K_2SO_4/H_2SO_4 anolyte at (a)100, (b) 200, (c) 300, (d) 400, (e) 500, and (f) 600 mA cm^{-2}. No iR correction was applied. Source data for Supplementary Fig. 12 are provided as a Source Data file.

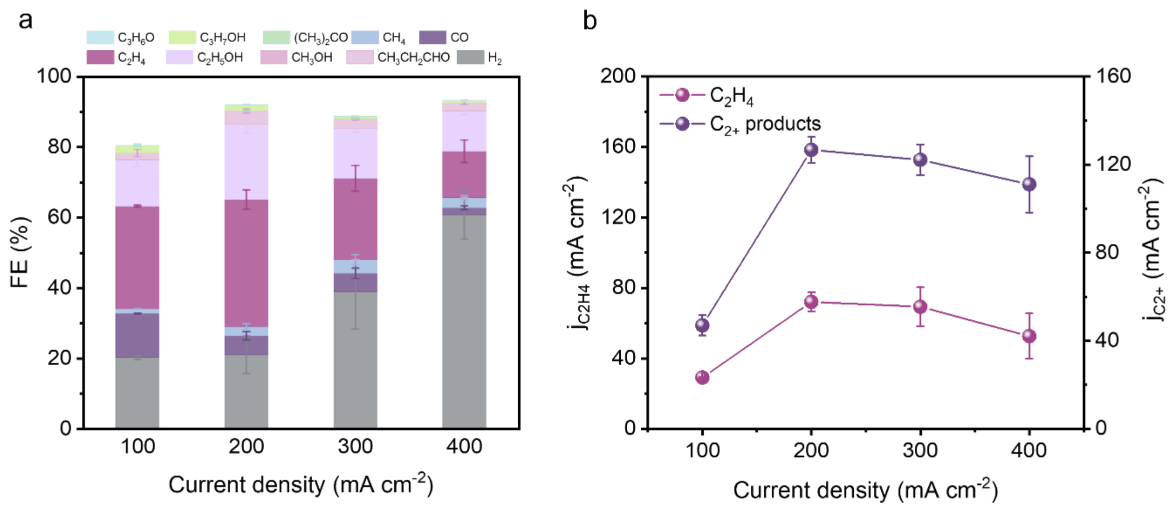

Supplementary Fig. 13 | CO_2R product distribution (a) and C_2H_4, C_{2+} products partial current of Cu_3N coated with Sustainion ionomer (Sus/Cu_3N) electrodes at different current densities in acid-fed MEA system in 0.5 M K_2SO_4/H_2SO_4 (ph~1) condition. No iR correction was applied. Source data for Supplementary Fig. 13 are provided as a Source Data file.

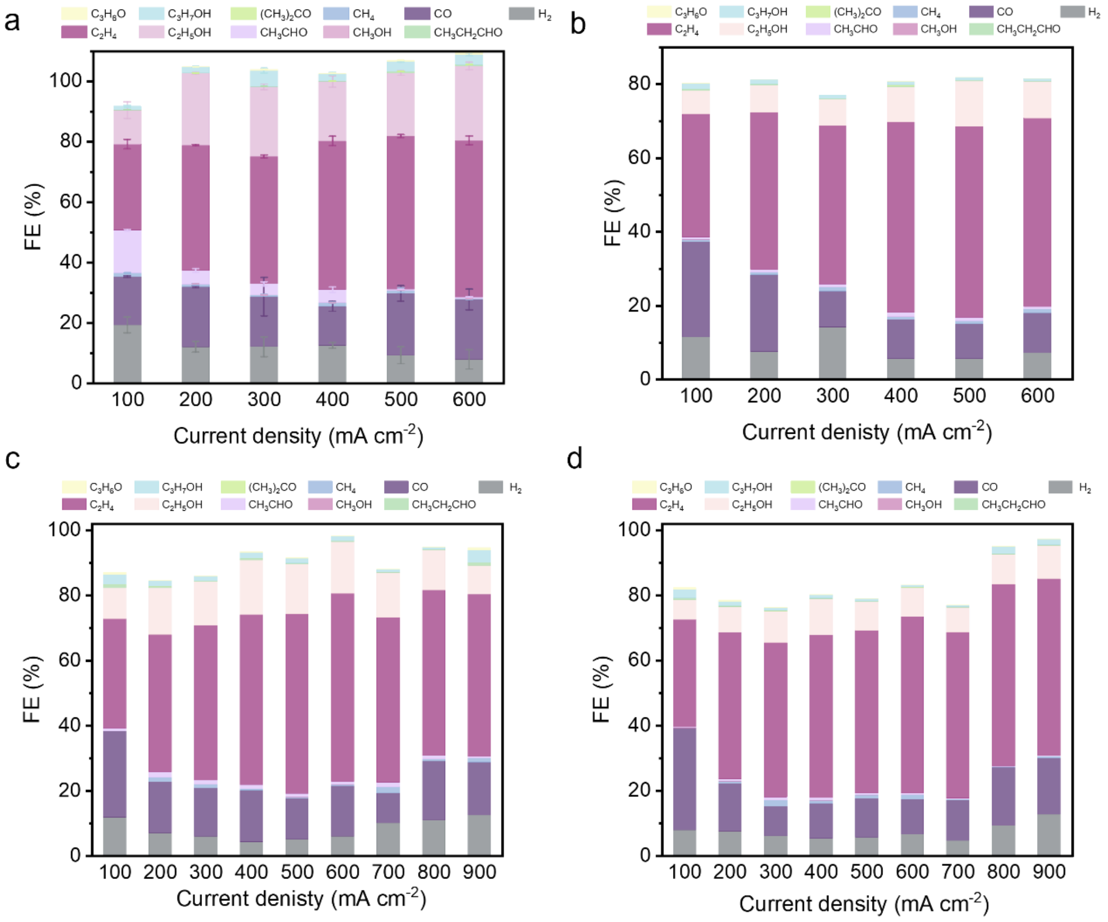

Supplementary Fig. 14 | CO_2R product distribution of CNCP electrodes at different current densities in acid-fed MEA system in a pH range from (a) ph 1.2, (b) ph 2.4, (c) ph 4.6 and (d) ph 6.3 conditions. No iR correction was applied. Source data for Supplementary Fig. 14 are provided as a Source Data file.

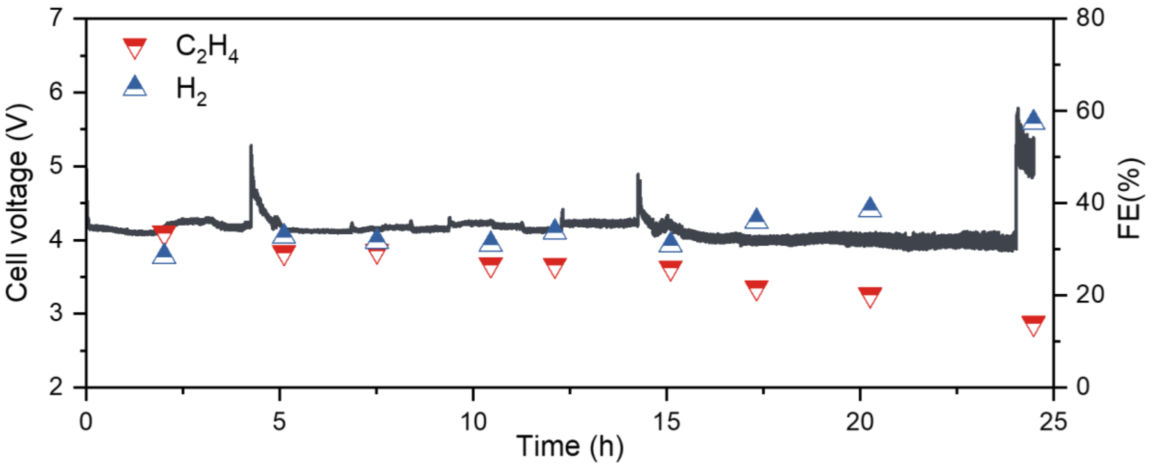

Supplementary Fig. 15 | The system stability performance of CO_2R to C_2H_4 on Sus/Cu_3N GDE in a scale-up MEA system at a constant current of 10 A. No iR correction was applied. Source data for Supplementary Fig. 15 are provided as a Source Data file.

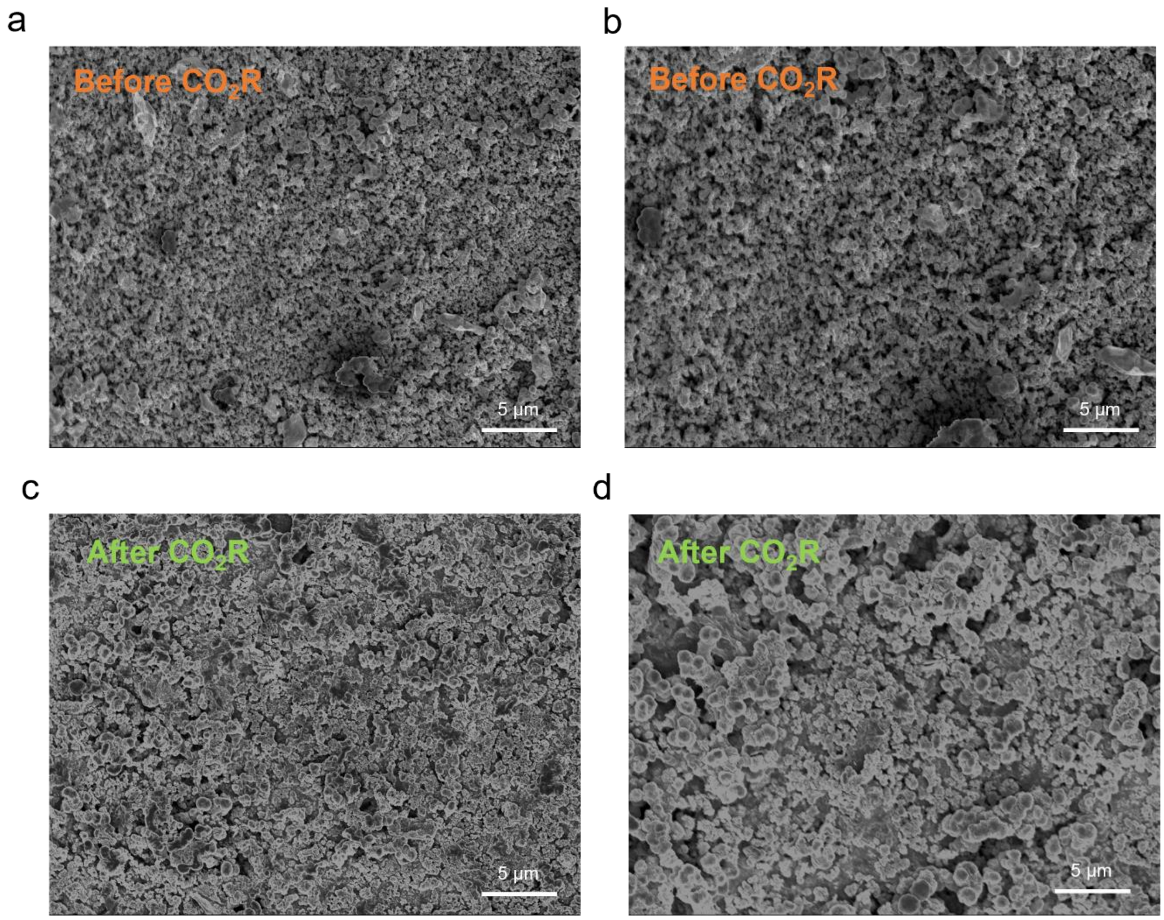

Supplementary Fig. 16 | Characterization of the CNCP electrodes before (a, b) and after (c, d) the extended CO_2R. From the comparison of the electrode surface morphology before and after the reaction, it can be seen that after a long-term stability test, there is no obvious structural change on the CNCP electrode surface, indicating the robust CNCP architecture.

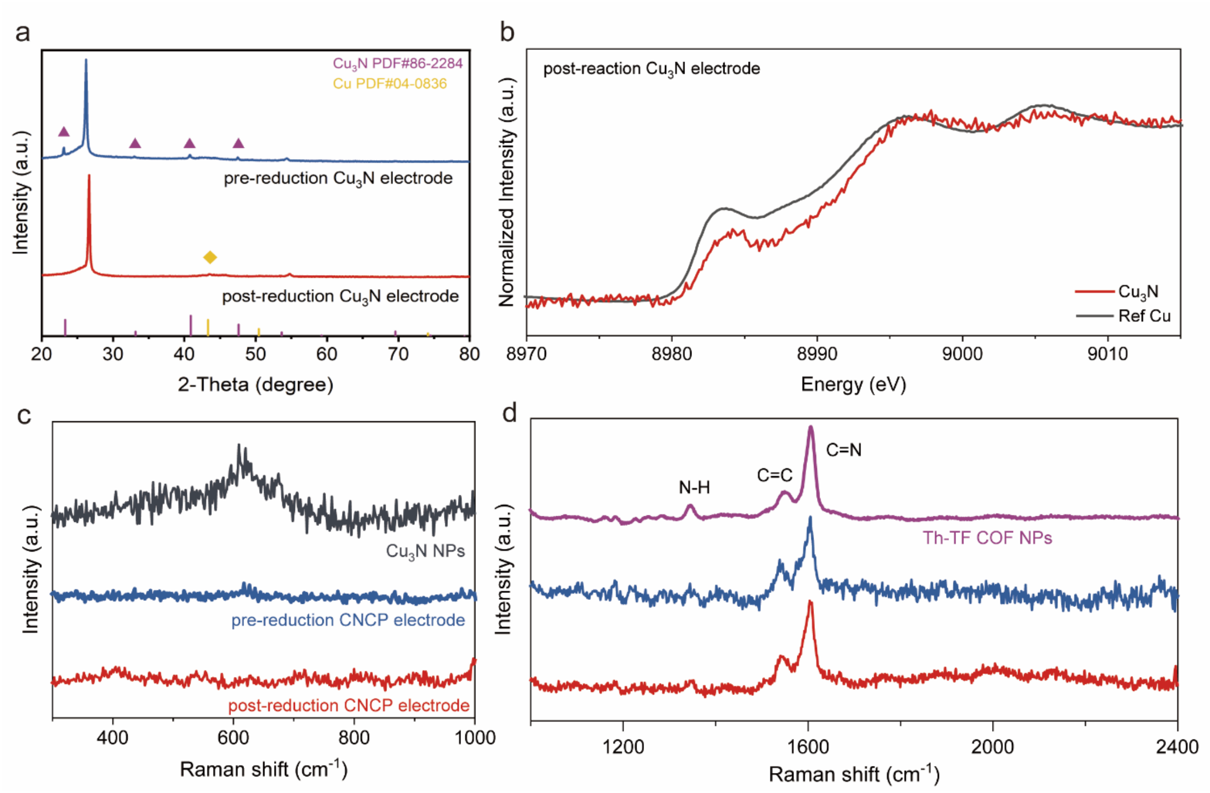

Supplementary Fig. 17 | Structural evolution of Cu_3N catalyst before and after CO_2RR. (a) XRD patterns of pristine Cu_3N with reflections at ~23.3°, 40.9°, and 47.6° (PDF#86-2284), which vanish after CO_2RR, while a broad peak at ~43-45° emerges from metallic Cu (111); peaks at ~25° and ~55° arise from the carbon paper. (b) Post-reaction Cu_3N shows a Cu K-edge XANES nearly identical to metallic Cu, with the edge aligned to Cu foil and a weakened white line, evidencing reconstruction into Cu^0 with minimal Cu-N coordination. (c) Raman spectra of Cu_3N nanoparticles, CNCP before, and after reaction, showing disappearance of the Cu-N band (~500-650 cm^{-1}) after CO_2RR. (d) Raman spectra of Th-TF COF nanoparticles, CNCP before, and after reaction, showing preserved bands at ~1620 cm^{-1} (C=N), ~1570 cm^{-1} (C=C), and ~1344 cm^{-1} (N-H), confirming COF stability during electrolysis. Source data for Supplementary Fig. 17 are provided as a Source Data file.

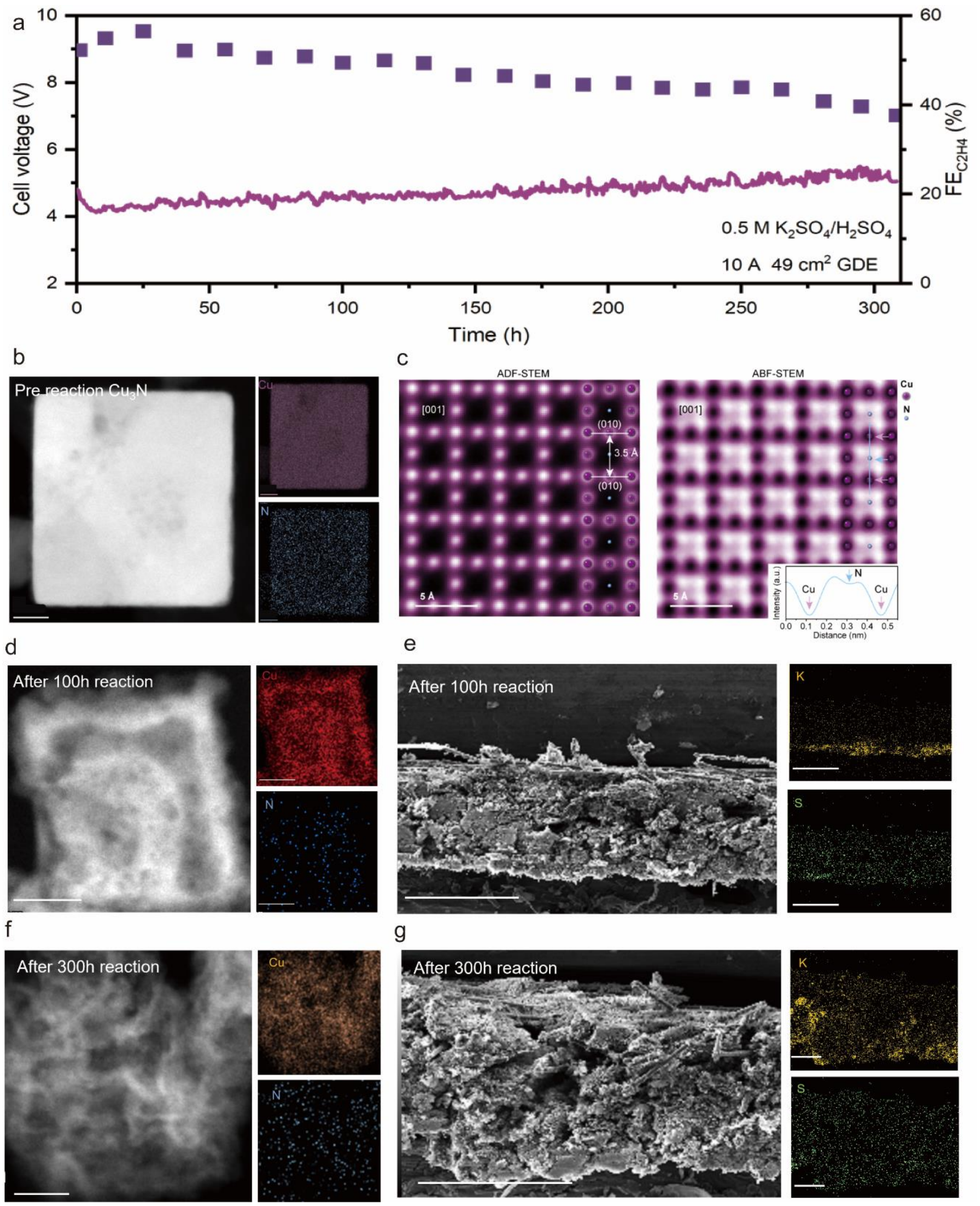

Supplementary Fig. 18 | Long-term stability and structural evolution of CNCP electrodes during CO_2 electroreduction. (a) Stability test of the CNCP electrode in 0.5 M K_2SO_4/H_2SO_4 at a constant current of 10 A (49 cm^2 GDE), showing cell voltage (purple squares, left axis) and C_2H_4 Faradaic efficiency (purple line, right axis) over 300 h. (b) STEM-EDS mapping of pristine Cu_3N, confirming uniform Cu and N distribution. Scale bars: 20 nm. (c) Atomic-resolution ADF- and ABF-STEM images of pristine Cu_3N along the [001] zone axis, resolving the ordered Cu

and N sublattices with an interplanar spacing of 3.5 Å. Scale bars: 20 nm. (d,f) TEM and EDS mapping after 100 h and 300 h electrolysis, respectively, showing progressive nitrogen depletion and reconstruction into Cu-rich phases. Scale bars in (d): 50 nm. Scale bars in (f): 20 nm. (e,g) Cross-sectional SEM-EDS after 100 h and 300 h, respectively. At 100 h, the GDL retains porosity with K and S signals localized near the catalyst layer, whereas after 300 h extensive electrolyte infiltration and loss of porosity are evident, with K and S penetrating deep into the GDL. Scale bars: 200 μm.

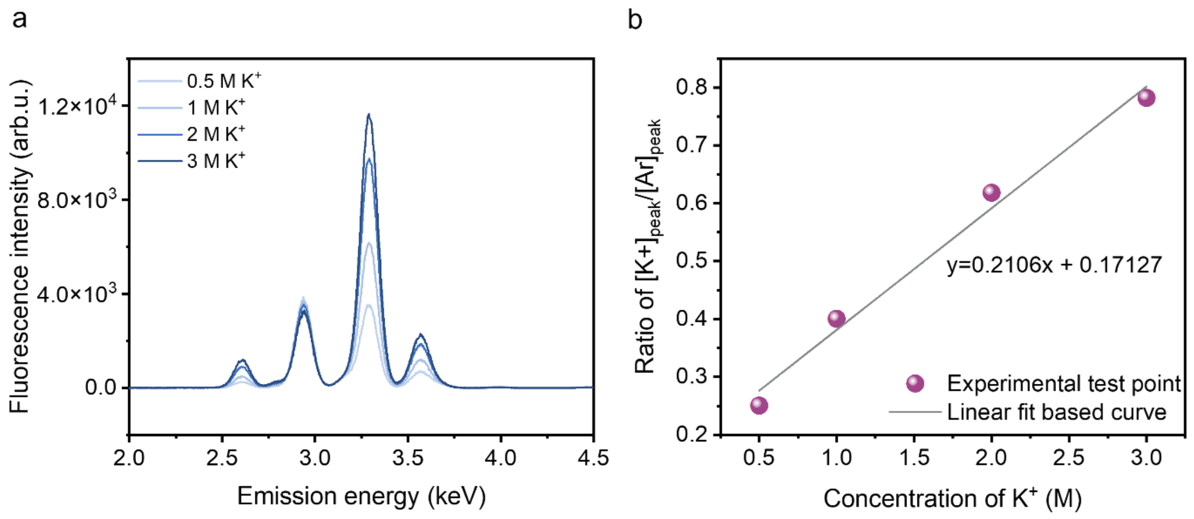

Supplementary Fig. 19 | a) XRF spectrum of Ar K, K Kα, and Kβ peaks across a K^+ concentration gradient from 0.5-3 M in K_2SO4/H_2SO4 (ph~3) solutions. b) A linear baseline correlating the K^+/Ar peak ratio to bulk K^+ concentration enabled calculation of absolute K^+ amounts. Source data for Supplementary Fig. 19 are provided as a Source Data file.

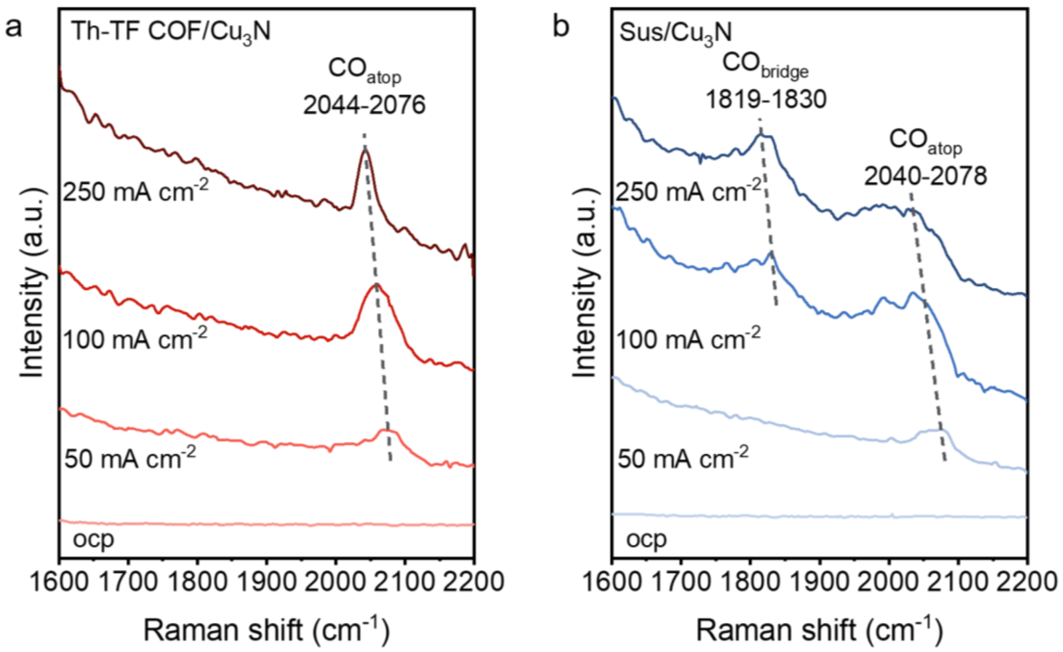

Supplementary Fig. 20 | In-situ Raman spectra in a 0.5 M K_2SO4/H_2SO4 (ph~3) solution for electrosynthesis of C_2H_4 over Th-TF COF (a) and Sustainion coated Cu_3N (b) catalysts at different current densities. No iR correction was applied. Source data for Supplementary Fig. 20 are provided as a Source Data file.

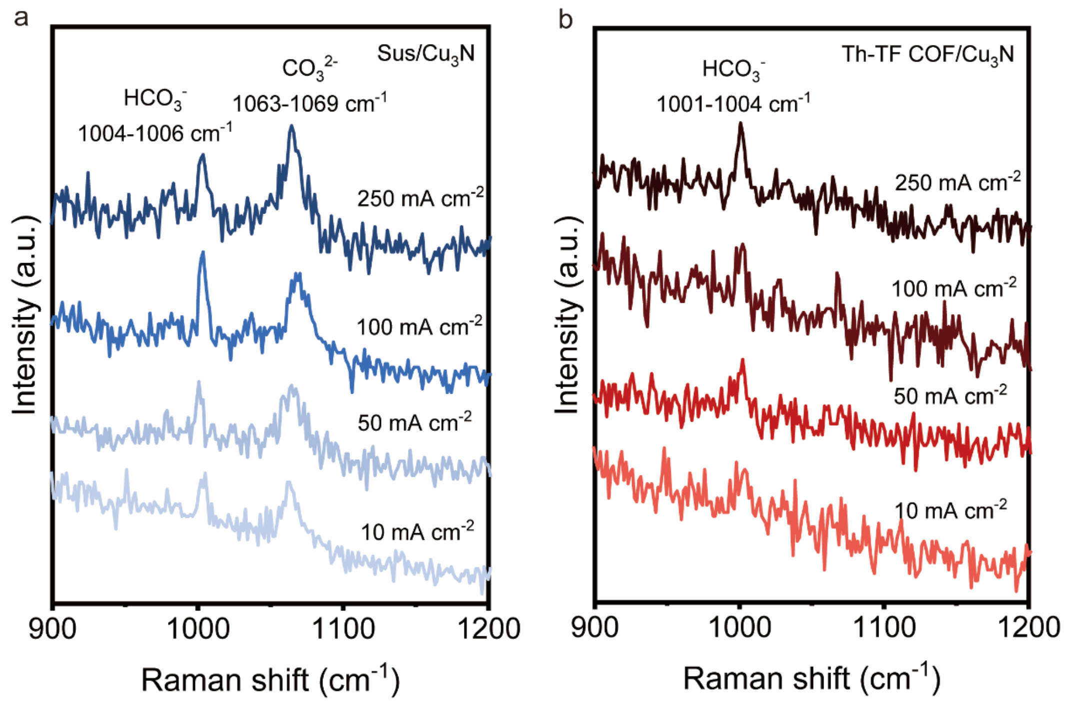

Supplementary Fig. 21 |In-situ Raman spectra of carbonate species during acidic CO_2 reduction. (a) Ionomer-coated Cu_3N (Sus/Cu_3N) electrode, where HCO_3^- (~1004-1006 cm^{-1}) and CO_3^{2-} (~1063-1069 cm^{-1}) bands emerge and intensify with current density (10-250 mA cm^{-2}), indicating progressive carbonate accumulation near the catalyst surface. (b) Th-TF COF/Cu_3N electrode, displaying only a weak HCO_3^- shoulder (~1001-1004 cm^{-1}) and no discernible CO_3^{2-} band across the same current range, evidencing suppressed carbonate formation and a less alkaline local environment. No iR correction was applied. Source data for Supplementary Fig. 21 are provided as a Source Data file.

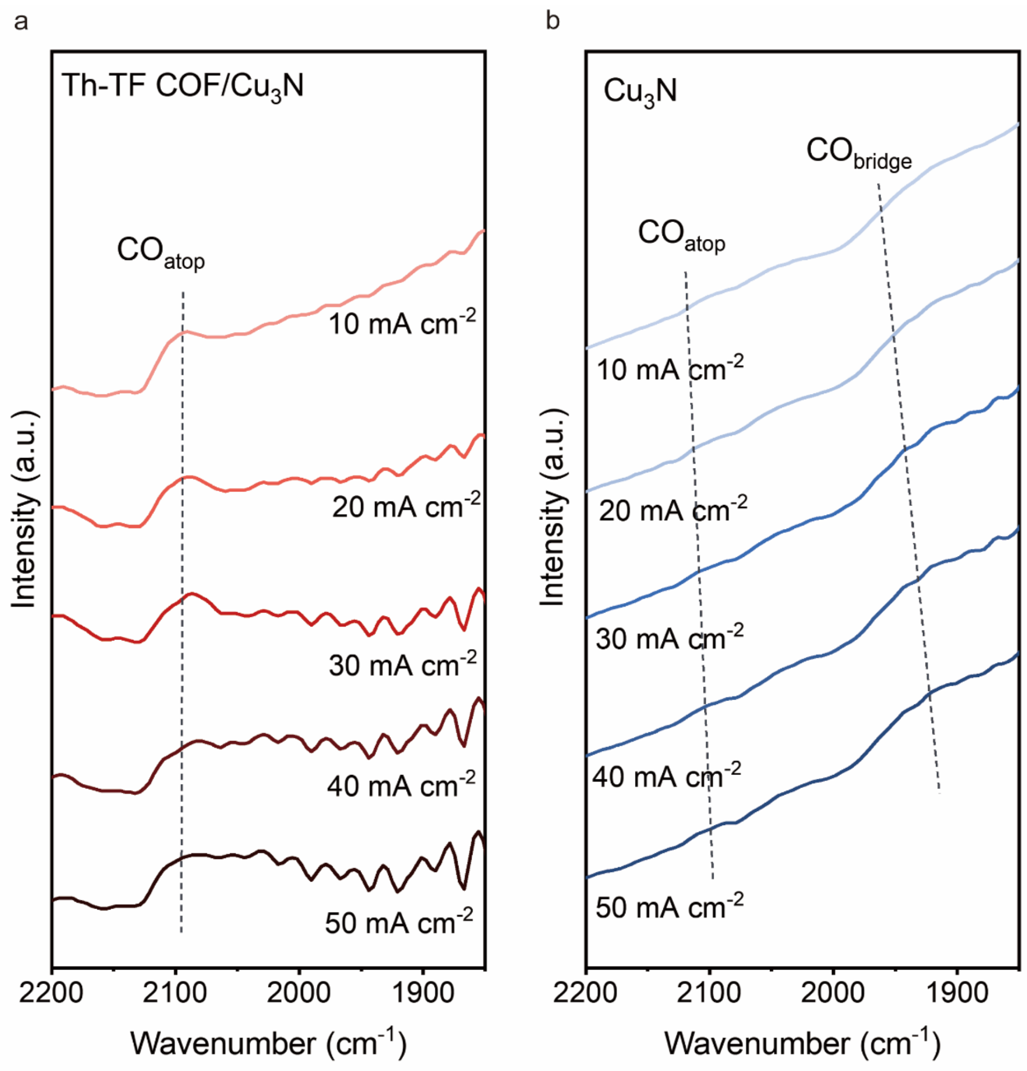

Supplementary Fig. 22 | In-situ ATR-FTIR spectra of CO adsorption during acidic CO_2 reduction. (a) Th-TF COF/Cu_3N electrode displaying a clear *COatop band (2040-2070 cm^{-1}) across current densities of 10-50 mA cm^{-2}. (b) Bare Cu_3N electrode predominantly showing *CObridge adsorption (1815-1830 cm^{-1}) under identical conditions. The contrast between *COatop and *CObridge configurations highlights the lower local pH environment sustained by the COF layer, consistent with Raman results. No iR correction was applied. Source data for Supplementary Fig. 22 are provided as a Source Data file.

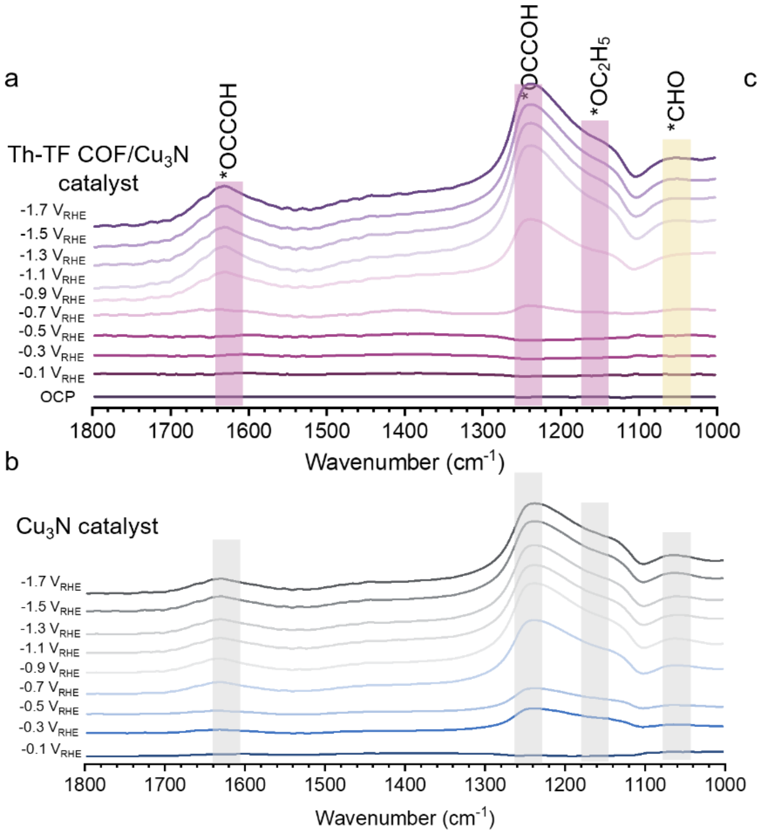

Supplementary Fig. 23 | In-situ ATR-SEIRS spectra recorded during acidic CO_2R on Th-TF COF/Cu_3N (a) and bare Cu_3N (b) catalysts from −0.3 to −1.7 V versus Ag/AgCl. c) Intenisty of *OCCOH during acidic CO_2R on Th-TF COF/Cu_3N and bare Cu_3N catalysts at the wavenumber range of 1,100 to 1,750 cm^{-1}. No iR correction was applied. Source data for Supplementary Fig. 23 are provided as a Source Data file.

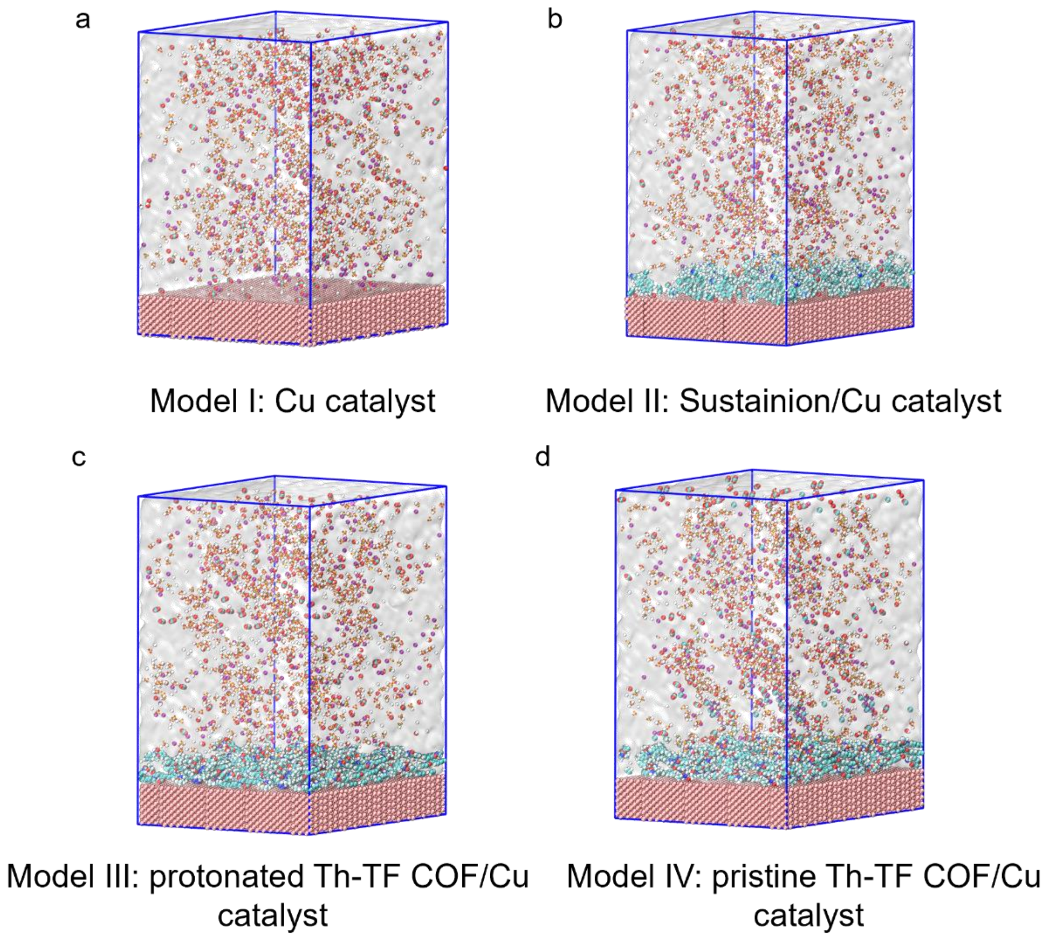

Supplementary Fig. 24 | We developed a simplified three-dimensional ion and molecular transport model to simulate the distribution of species near the catalyst surface during acidic CO_2R (0.5 M K_2SO_4/H_2SO_4; ph1; 500 mA cm^{-2}), to evaluate the ions (K^+ and OH^-) and CO_2 molecules regulation capabilities. Four models were compared: the bare Cu catalyst (Model I), Cu coated with a Sustainion ionomer (Sus/Cu, Model II), Cu coated with protonated Th-TF COF (Th-TF COF/Cu, Model III) and pristine Th-TF COF (Th-TF COF/Cu, Model IV). The simulation region was limited at distances (0-15 nm) near the catalyst layer, and a thin/porous coating layer (set as 2 nm) was employed between the catalyst surface and the electrolyte domain.

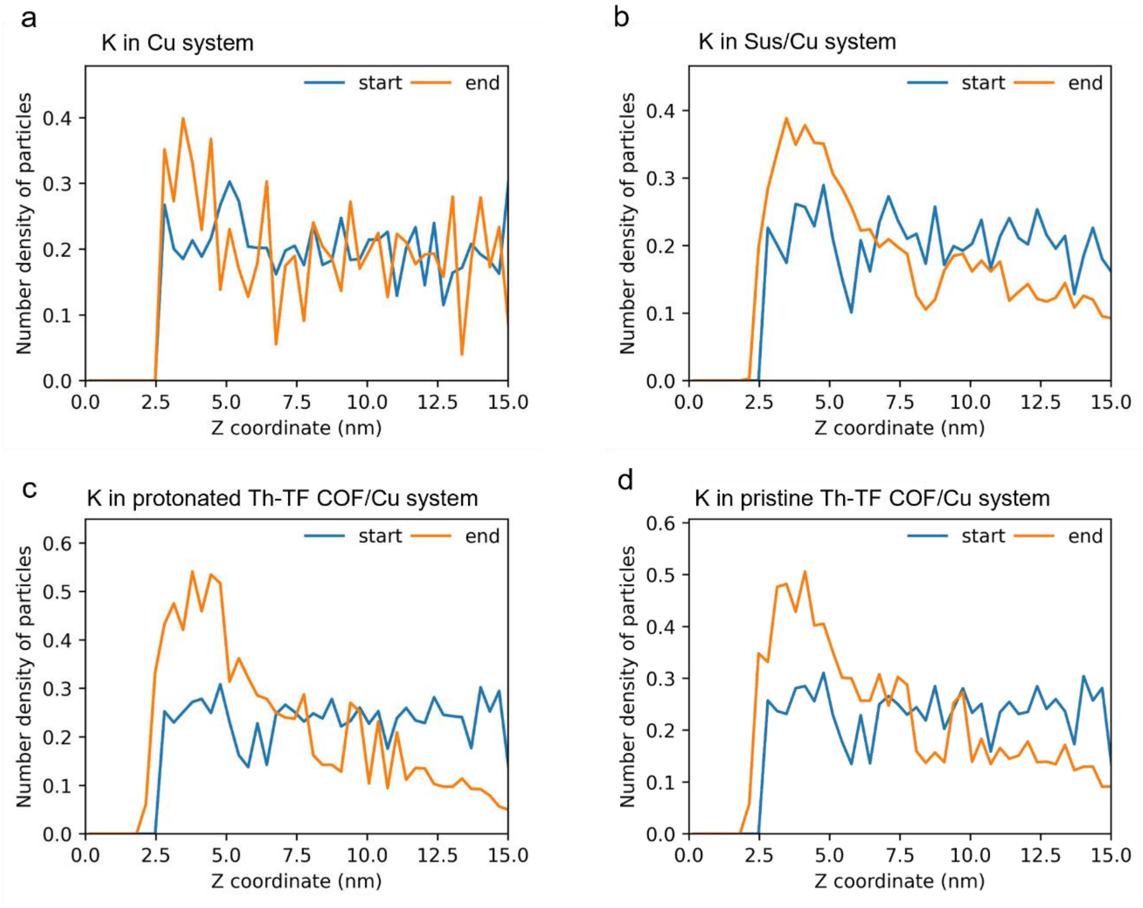

Supplementary Fig. 25 | K^+ density calculations in Cu (a), Sus/Cu (b), protonated Th-TF COF/Cu (c) and pristine Th-TF COF/Cu (d) systems. 0-2.5 nm was set as the distance from the bottom copper atom to the top copper atom, reflecting the thickness of the copper catalyst. Source data for Supplementary Fig. 25 are provided as a Source Data file.

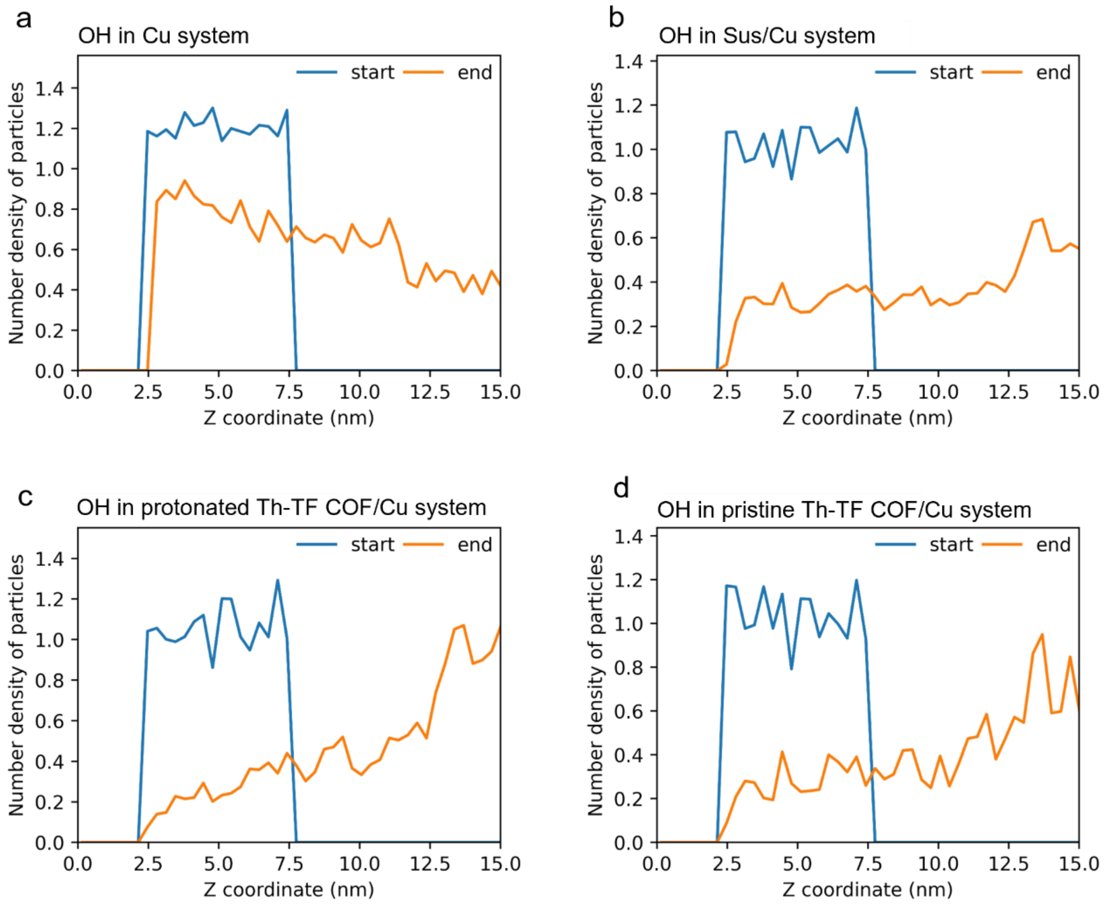

Supplementary Fig. 26 | Calculated OH⁻ density in Cu (a), Sus/Cu (b), protonated Th-TF COF/Cu (c) and pristine Th-TF COF/Cu (d) systems. 0-2.5 nm was set as the distance from the bottom copper atom to the top copper atom, reflecting the thickness of the copper catalyst. Source data for Supplementary Fig. 26 are provided as a Source Data file.

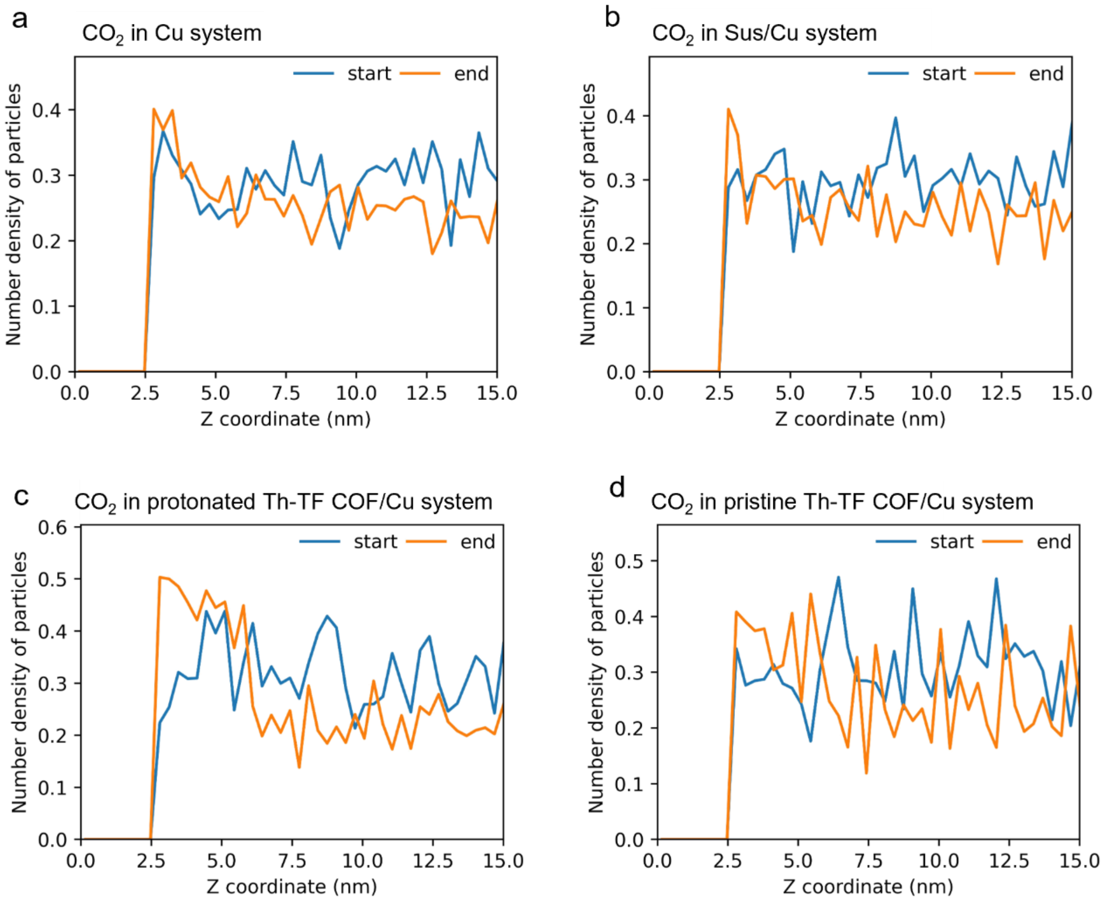

Supplementary Fig. 27 | Calculated CO_2 molecule density in Cu (a), Sus/Cu (b), protonated Th-TF COF/Cu (c) and pristine Th-TF COF/Cu (d) systems. 0-2.5 nm was set as the distance from the bottom copper atom to the top copper atom, reflecting the thickness of the copper catalyst. Source data for Supplementary Fig. 27 are provided as a Source Data file.

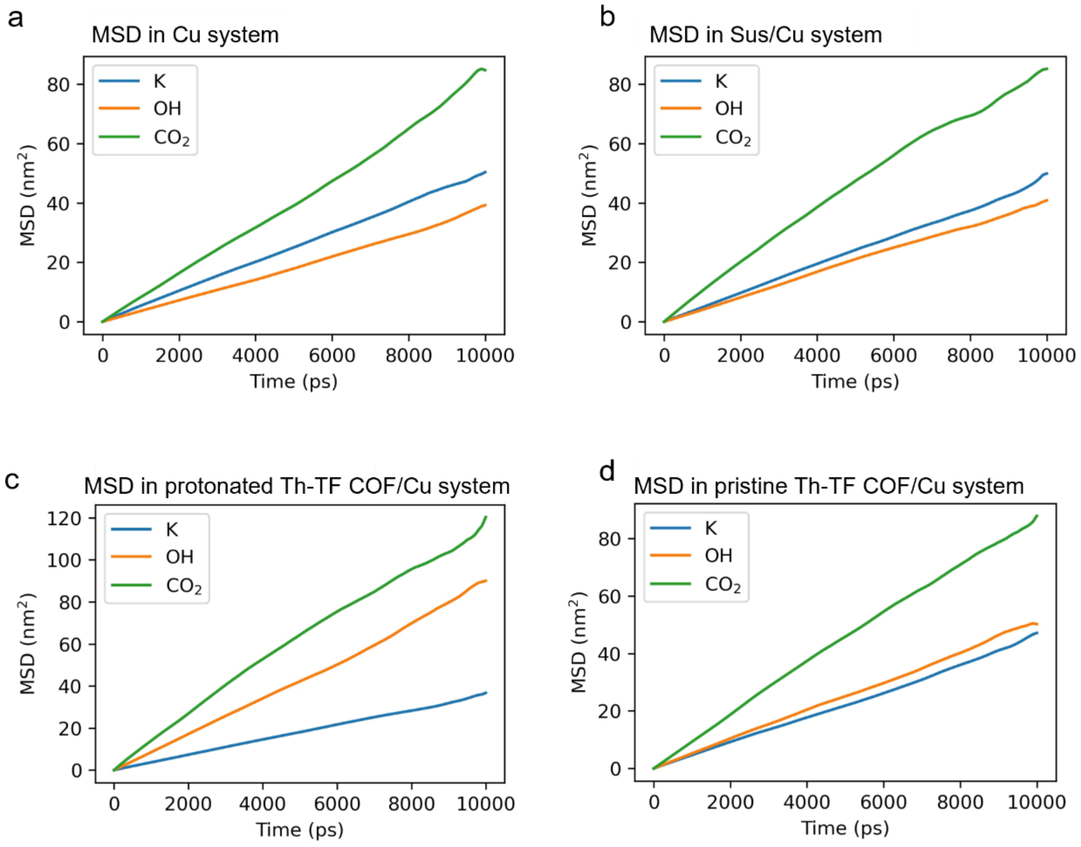

Supplementary Fig. 28 | Mean Square Displacement (MSD) calculations in Cu (a), Sus/Cu (b), protonated Th-TF COF/Cu (c) and pristine Th-TF COF/Cu (d) systems. Source data for Supplementary Fig. 28 are provided as a Source Data file.

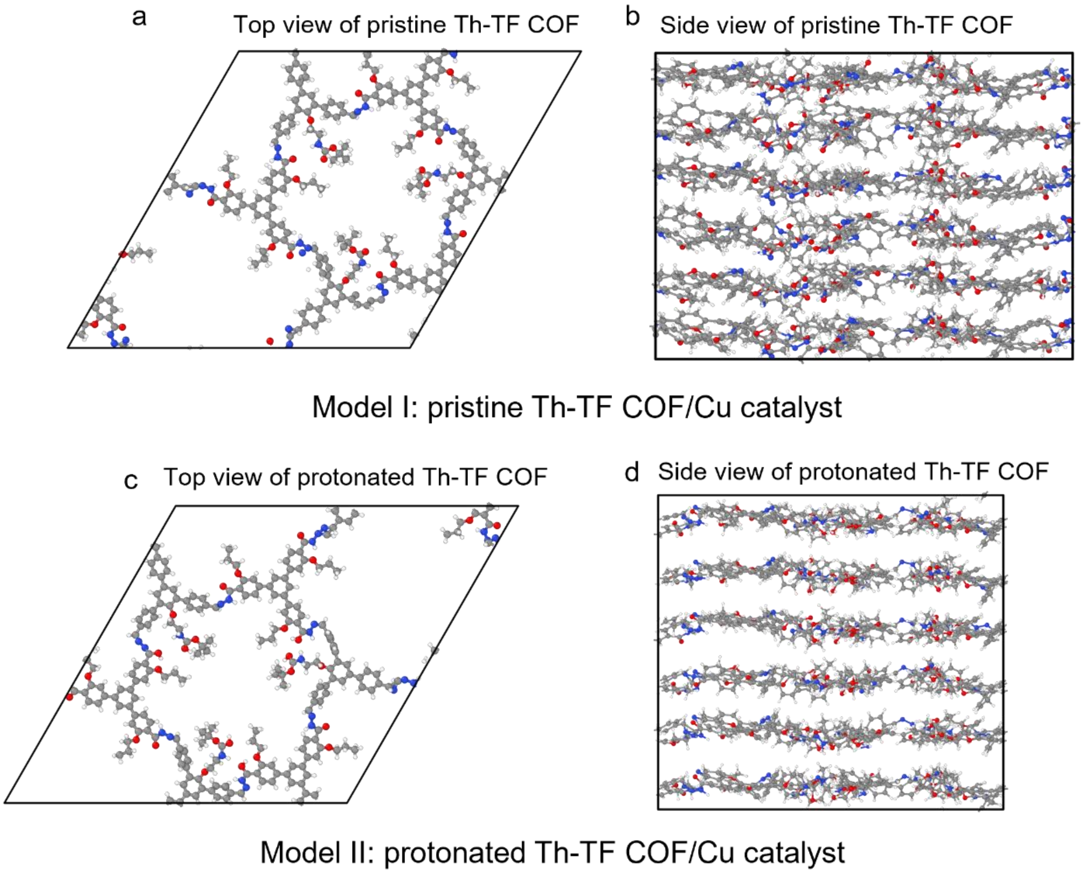

Supplementary Fig. 29 | To investigate ion interactions within the Th-TF COF framework, we simulated dynamic bidirectional flow through nanochannels of COFs. Two models were compared: pristine (Model I) and protonated (Model II) Th-TF COF catalysts. a) and b) show the top and side views of pristine Th-TF COF in simulated box. c) and d) show the top and side views of protonated Th-TF COF in simulated box.

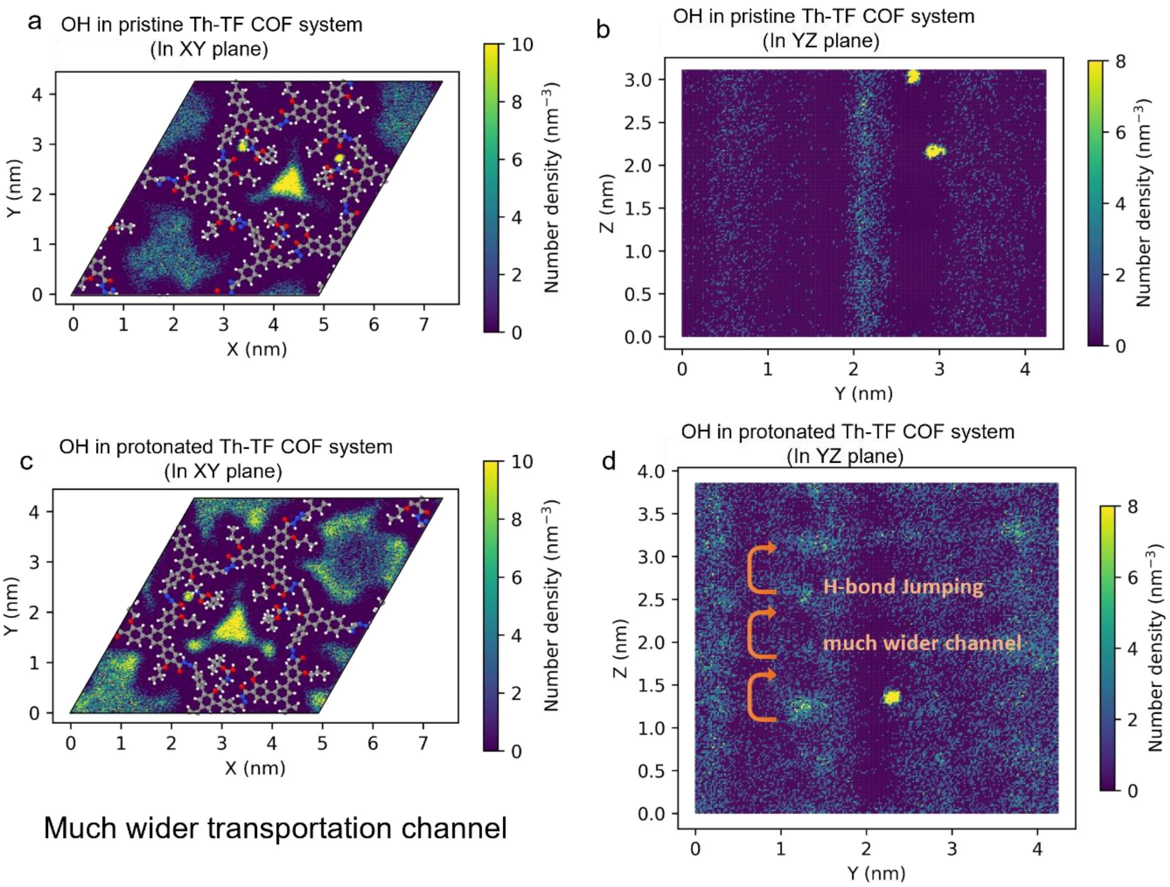

Supplementary Fig. 30 | OH density maps in the XY plane of pristine Th-TF COF/Cu (a) and protonated Th-TF COF/Cu (c) systems. The fluorescent part shows the interaction sites between OH ions and COF's molecular structure. The intensity of fluorescence reflects the strength of the interaction between OH^- ions and molecules. OH^- density maps in YZ plane of pristine Th-TF COF/Cu (b) and protonated Th-TF COF/Cu (d) systems. The simulation shows that OH^- ions can migrate along the COF nanochannels under the action of electric field and concentration gradient, among which the protonated Th-TF COF has a wider migration channel than the pristine one. Source data for Supplementary Fig. 30 are provided as a Source Data file.

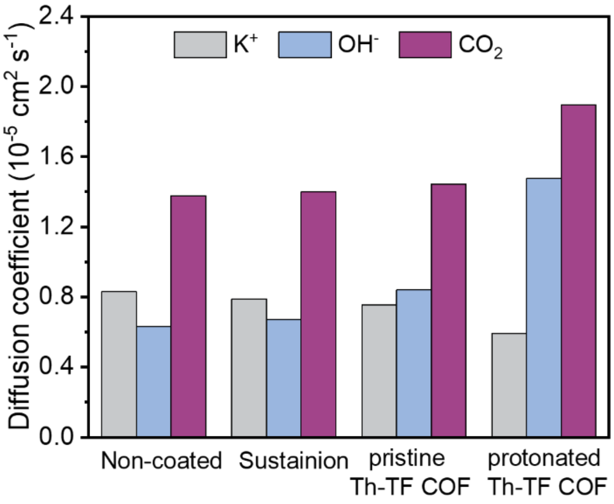

Supplementary Fig. 31 | Self-diffusion coefficient derived from MSD calculation of K^+ and OH^- and CO_2 for Cu, Sus/Cu, pristine Th-TF COF/Cu and protonated Th-TF COF/Cu models. Source data for Supplementary Fig. 31 are provided as a Source Data file.

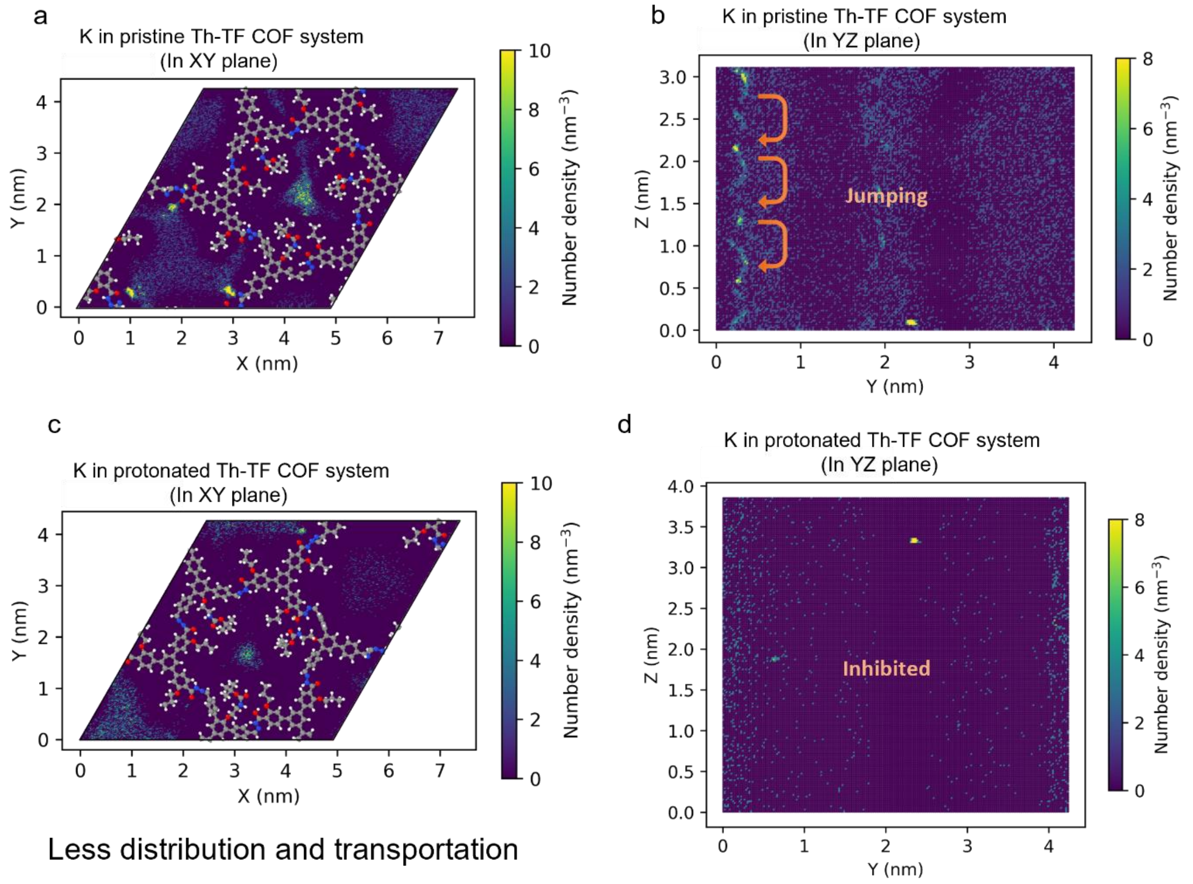

Supplementary Fig. 32 | K^+ density maps in XY plane of pristine Th-TF COF/Cu (a) and protonated Th-TF COF/Cu (c) systems. The fluorescent part shows the interaction sites between K ions and COF's molecular structure. The intensity of fluorescence reflects the strength of the interaction between K^+ ions and molecules. K^+ density maps in the YZ plane of pristine Th-TF COF/Cu (b) and protonated Th-TF COF/Cu (d) systems. The simulation shows that K^+ ions are rapidly enriched on the electrode surface through the COF channels under the action of electric field force and concentration difference in the early stage (results from density calculations discussed preveiously). The protonated COF forms an electric field of positive charge locally to inhibit the movement of K^+ ions in the YZ direction, thereby achieving the confinement effect on K^+ ions. Source data for Supplementary Fig. 32 are provided as a Source Data file.

No.	Time/h	FE_{CO}/%	FE_{H2}/%	FE_{CH4}/%	FE_{C2H4}/%	FE_{gas}/%
**1**	1	6.30	19.00	4.84	52.24	82.37
**2**	10	8.64	17.17	1.88	54.89	82.57
**3**	25	9.07	16.75	0.60	56.45	82.87
**4**	40	9.10	17.04	0.56	52.13	78.83
**5**	55	10.15	18.59	0.75	52.38	81.87
**6**	70	10.30	20.33	1.21	50.54	82.38
**7**	85	8.77	18.20	2.79	50.84	80.60
**8**	100	10.36	21.28	1.42	49.41	82.46
**9**	115	9.09	18.77	2.85	49.93	80.65
**10**	130	8.93	20.62	3.25	49.33	82.12
**11**	145	10.27	22.36	1.52	46.70	80.85
**12**	160	9.71	20.33	3.84	46.47	80.35
**13**	175	6.56	26.36	4.18	45.30	82.40
**14**	190	10.66	25.37	1.81	44.52	82.37
**15**	205	8.81	24.37	4.12	44.86	82.17
**16**	220	8.57	24.62	5.40	43.83	82.42
**17**	235	8.50	24.78	5.69	43.43	82.39
**18**	250	8.06	23.94	5.45	43.91	81.36
**19**	265	7.94	24.65	5.39	43.41	81.39
**20**	280	11.18	27.62	2.06	40.82	81.68
**21**	295	7.80	28.43	5.52	39.63	81.38
**22**	308	7.76	30.53	6.31	37.64	82.24

Supplementary Table 1 | The system stability performance of CO_2R to C_2H_4 on CNCP GDE in a scale-up MEA system at a constant current of 10 A.

No	Works	Devices	Selectivity	FE_{C2H4}	Stability current	Stability
**7**	Science 372, 1074-1078 (2021)[1]	flow	FE_{C2+}~50%	~20%	1.2 A	12h
**20**	Angew. Chem. Int. Ed. 62 (2023)[2]	flow	FE_{C2+}~70%	~35%	0.05A	5h
**9**	Nature Catalysis 5, 564-570 (2022)[3]	flow	FE_{C2+}~89%	~40%	0.5 A	4.5h
**22**	Nature Communications 15, 491 (2024)[4]	flow	FE_{C2+}~87%	~40%	0.6A	10h
**11**	Nature Synthesis 2, 403-412 (2023)[5]	flow	FE_{C2+}~75%	~40%	0.2 A	30h
**25**	Nature Communications 15, 4821 (2024)[6]	flow	FE_{C2+}~86%	~40%	0.9A	40h
**23**	Nature Communications 14, 1298 (2023)[7]	flow	FE_{C2+}~69%	~50%	0.15A	35h
**13**	Nature Communications 14, 2387 (2023)[8]	flow	FE_{C2H4}~53%	~53%	0.2 A	10h
**24**	Nature Nanotechnology 19, 311-318 (2023)[9]	flow	FE_{C2H4}~61%	~61%	0.3 A	12h
**12**	Nature Catalysis 6, 763-772 (2023)[10]	flow	FE_{C2+}~80%	~40%	0.1 A	155h
	**Our Work**	**MEA**	**FE_{C2+}~83%**	**~53%**	**10 A scale-up**	**300h**

Supplementary Table 2 | Comparison of this work with previous studies on the acidic electrocatalytic CO_2 to multi-carbon products at a similar electrolyte pH.

Reference

1 Huang, J. E. *et al.* CO_2 electrolysis to multicarbon products in strong acid. *Science* **372**, 1074-1078 (2021).

2 Nie, W., Heim, G. P., Watkins, N. B., Agapie, T. & Peters, J. C. Organic Additive-derived Films on Cu Electrodes Promote Electrochemical CO_2 Reduction to C_{2+} Products Under Strongly Acidic Conditions. *Angew Chem. Int. Ed. Engl.* **62**, e202216102 (2023).

3 Xie, Y. *et al.* High carbon utilization in CO_2 reduction to multi-carbon products in acidic media. *Nat. Catal.* **5**, 564-570 (2022).

4 Sun, M., Cheng, J. & Yamauchi, M. Gas diffusion enhanced electrode with ultrathin superhydrophobic macropore structure for acidic CO_2 electroreduction. *Nat. Commun.* **15**, 491 (2024).

5 Zhao, Y. *et al.* Conversion of CO_2 to multicarbon products in strong acid by controlling the catalyst microenvironment. *Nat. Synth.* **2**, 403-412 (2023).

6 Feng, J. *et al.* CO_2 electrolysis to multi-carbon products in strong acid at ampere-current levels on La-Cu spheres with channels. *Nat. Commun.* **15** 4821 (2024).

7 Zhang, J. *et al.* Accelerating electrochemical CO_2 reduction to multi-carbon products via asymmetric intermediate binding at confined nanointerfaces. *Nat. Commun.* **14**, 1298 (2023).

8 Cao, Y. *et al.* Surface hydroxide promotes CO_2 electrolysis to ethylene in acidic conditions. *Nat. Commun.* **14**, 2387 (2023).

9 Chen, Y. *et al.* Efficient multicarbon formation in acidic CO_2 reduction via tandem electrocatalysis. *Nat. Nanotechnol.* **19**, 311-318, (2024).

10 Fan, M. *et al.* Cationic-group-functionalized electrocatalysts enable stable acidic CO_2 electrolysis. *Nat. Catal.* **6**, 763-772, (2023).
