# Supplementary material for: Electrocatalytic CO2 reduction to ethylene in an acid-fed membrane electrode assembly at 10 A
Source: Nat Commun. 2025 Nov 28;16:10783. doi: 10.1038/s41467-025-65831-8 (PMC12663450; doi:10.1038/s41467-025-65831-8)
Supplement: Supplementary file 2 — Description of Additional Supplementary Files [file 41467_2025_65831_MOESM2_ESM.pdf]

Description of Additional Supplementary Files

File Name: Supplementary Data 1

Description: The cubic Cu catalyst ($Fm\bar{3}m$) forms $CuCu_{12}$ cuboctahedra with uniform Cu–Cu bonds of 2.53 Å. This structural model was used in molecular dynamics (MD) simulations to investigate K^+, OH^-, and CO_2 transport near the catalyst interface. The simulation cell measured 9.8 × 9.8 × 16.5 nm^3, with periodic boundaries in x/y and non-periodic in z. A ~2 nm-thick Cu (100) slab supported a 14 nm electrolyte layer comprising 216 OH^-, 108 CO_2, 208 K^+, 1400 H^+ (pH ≈ 2), 700 SO_4^{2-}, and 39,600 H_2O molecules. All species except TIP3P water were described by GAFF, with RESP charges for CO_2, OH^-, and SO_4^{2-}, and +1 charges for K^+/H^+. Long-range interactions were treated using Lennard-Jones 12–6 and PME (1.0 nm cutoff). Simulations included energy minimization (2×10^2 kJ mol^{-1} nm^{-1}), NpT pre-equilibration (298 K, 10 ns, 2 fs timestep), and NVT production (298 K, 10 ns) under 0.5 A cm^{-2} effective current density.

File Name: Supplementary Data 2

Description: The molecular structure of the commercial Sustainion ionomer used as an interfacial overlayer on the Cu catalyst. This model was employed in MD simulations (cell size ~9.8 × 9.8 × 18 nm^3) to probe the transport behavior of K^+, OH^-, and CO_2 near the Sus/Cu interface. The system contained a 2 nm Sustainion layer atop a 2 nm Cu (100) slab and a 14 nm electrolyte layer consisting of 216 OH^-, 108 CO_2, 208 K^+, 1400 H^+ (pH ≈ 2), 700 SO_4^{2-}, and 39,600 H_2O molecules. Force fields and charges followed GAFF and RESP protocols as described for Supplementary Data 1. MD simulations were performed through energy minimization, 10 ns NpT equilibration, and 10 ns NVT production at 298 K under an effective current density of 0.5 A cm^{-2}.

File Name: Supplementary Data 3

Description: The Th-TF COF molecular structure used for interfacial ion/molecules transport simulations. The Th-TF COF consists of a hydrazone-linked ($R_1R_2C{=}N{-}NH{-}R_3$) framework functionalized with amine and oxygen-containing alkyl groups. In MD simulations (cell size ~9.8 × 9.8 × 18 nm^3), a 2 nm COF layer was supported on a 2 nm Cu (100) slab, covered by a 14 nm electrolyte region containing 216 OH^-, 108 CO_2, 208 K^+, 1400 H^+ (pH ≈ 2), 700 SO_4^{2-}, and 39,600 H_2O molecules. Force fields were based on GAFF with RESP charges for COF, CO_2, OH^-, and SO_4^{2-}, and +1 for K^+/H^+. The simulations involved steepest-descent minimization, 10 ns NpT equilibration, and 10 ns NVT production at 298 K under 0.5 A cm^{-2} current density.

File Name: Supplementary Data 4

Description: The protonated Th-TF COF molecular structure under acidic conditions. In this model, the C=N−NH and C–N groups undergo spontaneous protonation, forming $-NH_2^+$ sites that facilitate hydrogen-

bond-mediated OH^- transport. The protonated COF layer (2 nm) was placed atop a 2 nm Cu (100) slab in an ~9.8 × 9.8 × 18 nm^3 simulation cell, covered by a 14 nm electrolyte layer with the same composition as described above. All non-water species were modeled using GAFF with RESP or unit charges as appropriate. MD simulations followed the same three-stage protocol (energy minimization, 10 ns NpT, and 10 ns NVT runs) at 298 K and 0.5 A cm^{-2} effective current density to yield quantitative interfacial transport trajectories.
